# HGDP and HapMap Analysis by Ancestry Mapper Reveals Local and Global Population Relationships

**DOI:** 10.1371/journal.pone.0049438

**Published:** 2012-11-26

**Authors:** Tiago R. Magalhães, Jillian P. Casey, Judith Conroy, Regina Regan, Darren J. Fitzpatrick, Naisha Shah, João Sobral, Sean Ennis

**Affiliations:** 1 School of Medicine and Medical Science, University College Dublin, Dublin, Ireland; 2 National Children’s Research Centre, Our Lady’s Childrens Hospital Crumlin, Dublin, Ireland; 3 Instituto Gulbenkian de Ciência, Oeiras, Portugal; 4 Clique Research Cluster, School of Computer Science and Informatics, University College Dublin, Dublin, Ireland; 5 Complex and Adaptive Systems Laboratory, and Conway Institute of Biomolecular and Biomedical Research, University College Dublin, Dublin, Ireland; University of Uppsala, Sweden

## Abstract

Knowledge of human origins, migrations, and expansions is greatly enhanced by the availability of large datasets of genetic information from different populations and by the development of bioinformatic tools used to analyze the data. We present Ancestry Mapper, which we believe improves on existing methods, for the assignment of genetic ancestry to an individual and to study the relationships between local and global populations. The principle function of the method, named Ancestry Mapper, is to give each individual analyzed a genetic identifier, made up of just 51 genetic coordinates, that corresponds to its relationship to the HGDP reference population. As a consequence, the Ancestry Mapper Id (AMid) has intrinsic biological meaning and provides a tool to measure similarity between world populations. We applied Ancestry Mapper to a dataset comprised of the HGDP and HapMap data. The results show distinctions at the continental level, while simultaneously giving details at the population level. We clustered AMids of HGDP/HapMap and observe a recapitulation of human migrations: for a small number of clusters, individuals are grouped according to continental origins; for a larger number of clusters, regional and population distinctions are evident. Calculating distances between AMids allows us to infer ancestry. The number of coordinates is expandable, increasing the power of Ancestry Mapper. An R package called Ancestry Mapper is available to apply this method to any high density genomic data set.

## Introduction

Human genetic diversity is a fundamental question in biology, relevant to population genetics, and genome wide association studies. Studies aiming to identify causal disease alleles increase power by including multi-ethnic designs while taking stratification into account – as reported among others by Haiman et al [1].

Human diversity and ancestry assignment have been studied by two main methodologies: clustering and principal component analysis (PCA) [2]. In clustering, individuals are placed into groups based on similarities of SNP frequencies. Frappe [3] and Structure [4] are two such methodologies. Principal Component Analysis (PCA) reduces the information contained in SNP frequencies to components, which capture most genetic variability; one recent and popular methodology is Eigensoft [5]. Typically individuals are separated into distinct groups by plotting components against each other. This approach is data-set dependent because principal components vary depending on the diversity and number of samples.

In this work we present Ancestry Mapper (AM), a new methodology to analyze ancestry. Each individual is characterized by an Ancestry Mapper Id (AMid) which consists of a set of coordinates, each being a measure of similarity to a particular reference population. Distinctive characteristics of AM include: 1) reducing the information needed to characterize an individual from several thousand SNPs to a small, fixed set of numbers, 2) providing built-in ancestry and admixture information, easily assigning continental and regional ancestry, 3) being amenable to clustering with no need to include individuals of known ancestry for cluster labeling, 4) producing barcodes for populations indicating inter and intra-population similarity, 5) assessing the degree of isolation of populations, 6) being data set independent and allowing for inter data set comparisons, and 7) providing global and regional information simultaneously. In Ancestry Mapper each individual is characterized by a small, fixed and meaningful set of numbers, which is comparable across data-sets and allows individuals to be assigned to their genetic ancestry.

Ancestry Mapper Ids (AMids) are produced by comparing each individual against population references. We chose the Human Genome Diversity Project (HGDP) as the basis of population references. HGDP is inspired in the work of Cavalli Sforza, who used genetics and linguistics to study populations [6]. HGDP is represented by 51 populations which provide world-wide coverage [7]–[8] and have been used extensively as population identifiers [9]–[11]. Ancestry Mapper also draws inspiration from the representation of genomes as variant calls compared to a reference genome, such as the Variant Call Format (vcf), a standard way to represent whole genomes [12].

We applied AM to a joint dataset of HGDP and HapMap to examine global and regional relationships, exploring intra and inter-population heterogeneity, isolation and admixture. The global relationships provide insights about continental groups while information on local relationships were obtained by AMIds of geographical neighbors, identifying the main reasons for similarities and subtle differences (e.g., proximity to a North/South gradient in East Asia).

The AMids characterizes individuals by a vector of fixed length (51) with values in a similar range, thereby making it optimal for clustering algorithms. We clustered the AMids of HGDP and HapMap using a robust method, PAM – Partition Around Medoids [13]. As expected individuals of similar origins clustered together but we also observe individuals from the same population in different clusters, showing intra-population heterogeneity, and individuals from neighboring populations clustering together, suggesting similarity and gene flow. We observe a recapitulation of human migrations: a small number of clusters, reflect the continental blocks; when the number of clusters is increased details on ancestry emerge, including clusters composed exclusively of individuals of a single population.

## Results

### References for Ancestry Mapper

The HGDP data set is composed of 938 individuals of 51 populations, genotyped using the Illumina platform (644,285 SNPs) and is available at http://hagsc.org/hgdp/files.html [7]. The HapMap data set is composed of 1,184 individuals of 11 populations, 1,654,989 SNPs and is available at http://hapmap.ncbi.nlm.nih.gov [10]. We merged the two datasets and selected SNPs with missing calls inferior to 0.01%, resulting in 2,227 individuals and 289,160 SNPs (see material and methods).

Ancestry Mapper uses a single individual as the reference for each HGDP population; therefore 51 references form the basis of AM. We selected the references by calculating the Euclidean distances between individuals in each population using all selected 289,160 SNPs and then choosing the individual with the smallest median of the distances to all other members.

**Figure 1:**
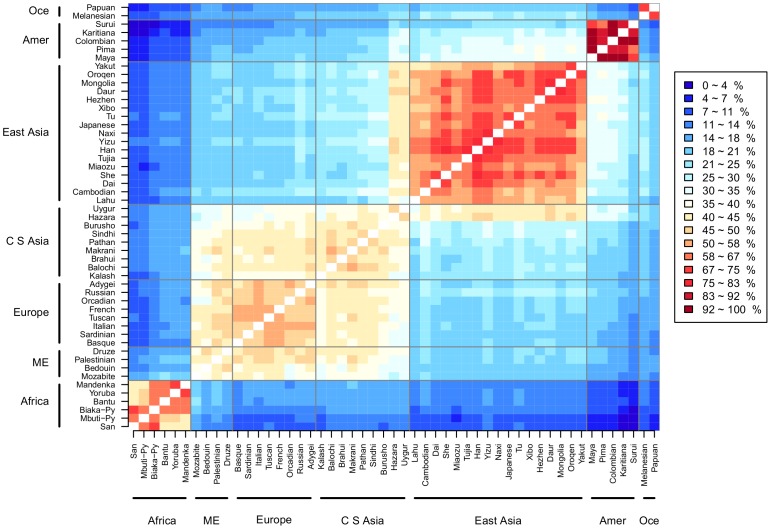
Distances between Ancestry Mapper references. Each reference is the individual which is the best representative of the HGDP population. The pair-wise combinations between the 51 references are plotted. The data is normalized so that 100 is given to the most similar pair. Vertical and horizontal lines separate the references into large continental groups: Africa, North-Africa/Middle East, Europe, Central South Asia, Eastern Asia and Oceania. Five large blocks of similarity are visible: 1) Africa, 2) NA/ME, Europe and Central-South Asia, 3) EA, 4) Americas, and 5) Oceania. The biggest block is composed of NA/ME, Europe, and CSA corresponding to the Indo-european continental group; within this large block the European references show the greatest similarity. The American block has the strongest similarity amongst its populations. The San and the Mbuti are the most distant to the other references.

Fig. 1 shows the distances between the 51 references. Our analysis visually identifies five blocks: (1) sub-Saharan Africa, (2) North Africa (NA), Middle East (ME), Europe and Central-South Asia (CSA), (3) East Asia (EA), (4) America, and (5) Oceania. Three different sub-blocks are distinguishable within the NA-ME/Europe/CSA block. The references within the Americas show the highest intra-block similarity, particularly the pairs Colombia/Maya and Colombia/Karitiana (an indigenous population living in Amazônia). The blocks with the least similarity to one another are Africa/Oceania, and Africa/Americas. The Mbuti Pygmies and the San show the largest distances to the other references.

The main blocks are also clear in an hierarchical clustering of the 51 individuals selected as references (Fig. S1). The first branch separates the African from non-African populations, with other branches corresponding to the major continental blocks.

These findings illustrate that the references accurately represent the relationships between populations and support the use of references as a world-wide genomic ancestry map.

### AMids – Characterizing each Individual by 51 Coordinates

**Figure 2:**
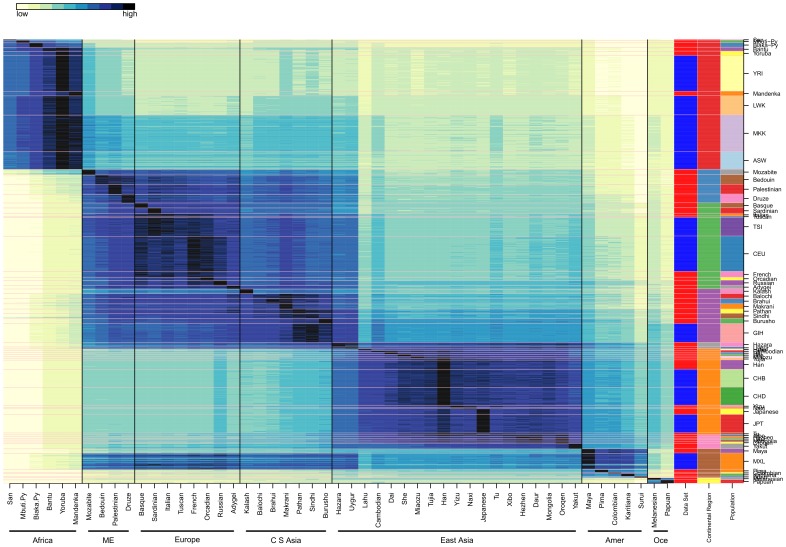
AMids for HGDP and HapMap. The normalized AMids of HGDP and HapMap individuals represent the genetic similarity to each of the 51 HGDP references (on the x axis); this Figure is a visual representation of Table S1. The individuals from the same population are stacked together. The second to last right column indicates continental regions. Blocks of regions are visible: 1) Africa, 2) North Africa, Middle East, Europe and Central South Asia, 3) Eastern Asia, 4) America, and 5) Oceania. Some populations are more isolated and include the San, both Pygmies, Mozabite, Kalash, Yakut, Surui, Pima, Colombian, Karitiana, Melanesian, and Papuan. Other populations show strong similarity with at least one other population (see for example Italian and French). The San, the Mbuti Pygmies and the Surui are the most distant to the majority of all others.

The Ancestry Mapper Id (AMid) is the Euclidean distances of an individual to the 51 references (one per HGDP population); for visualization purposes we normalize AMids (see material and methods). Normalized AMids for HGDP and HapMap individuals are shown in Fig. 2 and their values provided in Table S1 (supplementary materials).

**Figure 3:**
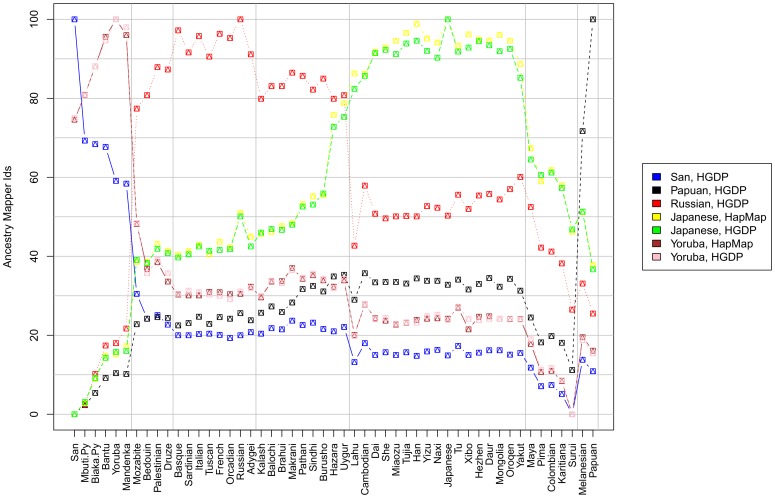
AMids of HGDP and HapMap individuals. AMids correspond to 51 references, placing every individual in a genomic map. For all individuals the highest AMid is the reference to their own population. For example, the San individual shows the highest AMid for the San reference, second for the Mbuti Pygmies, and third for the Biaka Pygmies, with all sub-saharan African AMids much higher than all others, indicating the big genetic difference of sub-sahara Africa to the rest of the world. Information on ancestry can be extracted from the barcode composed of all the AMids with valuable information present in the highest AMId but also on the others. The degree of genetic isolation can be assessed based on the difference of highest to second highest AMId. The Russian individual has high AMids for many references, especially European ones, but also CSA and ME. Conversely, both San and Papuan are shown to be quite distant from any other HGDP reference, with the second highest AMid for both of them less than 75. Individuals from the same ancestry in both HGDP and HapMap show the same results; observe the similarities between Japanese HGDP/HapMap and Yoruba HGDP/HapMap.

AMids of seven individuals of different origins are shown in Fig. 3. Information on ancestry is directly obtained by it. For example, the San individual is most similar to the San reference. Although relatively distant, the next most similar reference is the Mbuti Pygmy, with AMIds for both Pygmies very similar. San and Pygmies are hunter gatherers (HG), and their relative genetic closeness is expected. The African agriculturalists references are the next closest. All other references are much more distant.

AMids of individuals of the same origin, but from different datasets (HGDP/HapMap) show similar results. Both Yoruba have the highest AMid for Yoruba, high values for African references and much lower for other populations. The Japanese show the highest AMid for the Japanese, high values for Asian references and much lower for others. In addition to exploring the ancestry of an individual, AM can be used to examine relative levels of population isolation: the relative isolation of the San and the Papuan is shown as they are the most similar to their own reference and have large distances to the second closest reference.

**Figure 4:**
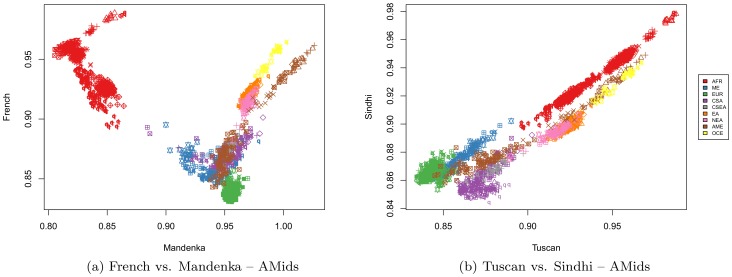
Bi-plots of AMids. (4a) Mandenka vs. the French AMid. The continental regions are clearly separated, with the Africans well apart along the Mandenka axis, while the Europeans are the closest to the French reference. Individuals from the Americas show a wide range of French AMid. All Oceanic individuals and some Native-American, placed in the upper-right quadrant, are distant both from the African and the European references. The spread of the Native-American along the French AMid indicates the diversity of those populations, especially of Mexicans from HapMap. (4b) Tuscan vs. Sindhi AMids. Individuals plotted in the Tuscan vs. Sindhi AMids show that these two AMids are well correlated, with individuals from Europe and CSA in the lower-left corner, indicating similarity. The continental regions are quite distinctive.

Bi-plots of one AMid against another produce information centered around specific populations. French vs. Mandenka AMids separates the world continental regions quite clearly (see Fig. 4a), while plotting Tuscan vs. Sindhi AMids shows CSA and European close to each other, with individuals from both populations in the quadrant of high reciprocal similarity (see Fig. 4b).

**Figure 5:**
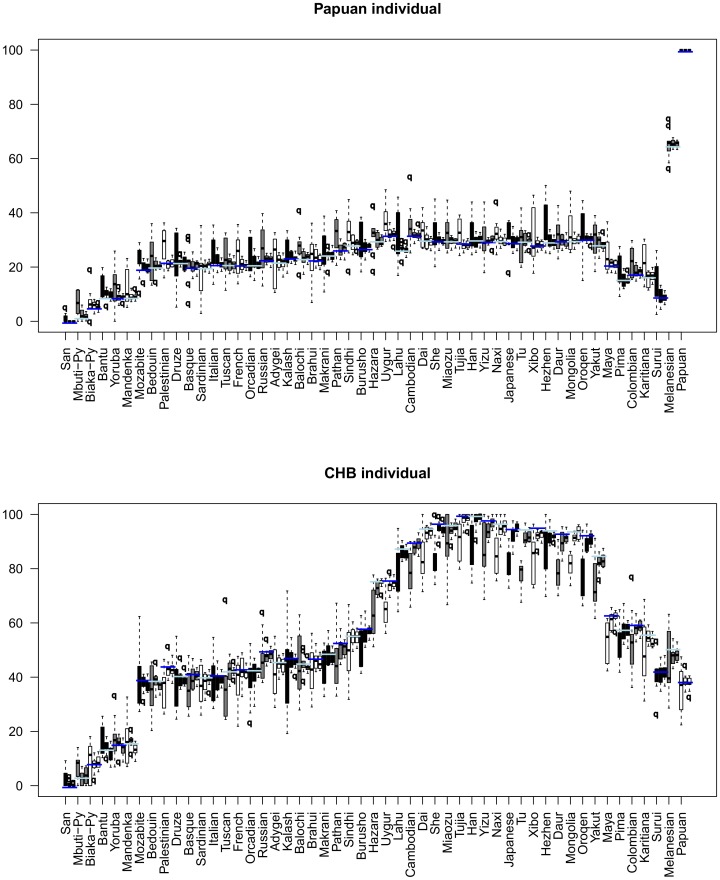
AMids of Papuan and Han calculated with 1,000, 10,000 and 20,000 SNPs. For each individual, we calculated 10 different sets of AMids using for each time a randomly selected set of SNPs. We used 1,000, 10,000 and 20,000 SNPs. There are 3 boxes for each reference in the order of 1,000/10,000/20,000 SNPs. The blue line represents the AMId when using the whole set of 289,160 SNPs (see Fig. 2). We observe that for 1,000 SNPs the variation is much bigger than for 10,000 or 20,000. In any case, the highest and the lowest AMids are quite separated. The barcode for the AMids is the same, even for 1,000 SNPs.

AM is dataset-context independent, in the sense that AMids are always the same, regardless of other elements in the dataset. However, AMids could be dependent on the number of SNPs used to calculate the distance to the references or on the actual SNPs used. To investigate this issue we calculated AMids for several individuals using randomly selected SNPs. We observe that for 1,000 SNPs the variance is quite substantial, decreasing when the number of SNPs is increased up to 20,000 SNPs (see Fig. 5). We calculated AMids for each individual using a different set of 20,000 randomly selected SNPs and the results are virtually indistinguishable from the original AMids (compare Fig. 2 with Fig. S2). This seems to indicate that AMids are robust and not only independent of other members of the dataset but also dataset independent. The SNPs we use had a high call rate and we have not extensively researched situations with lower call rates.

### Ancestry Mapper Highlights Human Migrations

From the combined data of HGDP and HapMap (see Fig. 2) we visually observe 5 blocks: 1) sub-Saharan Africa, 2) a large block containing North-Africa (NA), Middle East (ME), Europe and Central South Asia (CSA), 3) East Asia (EA), 4) Americas, and 5) Oceania.

**Figure 6:**
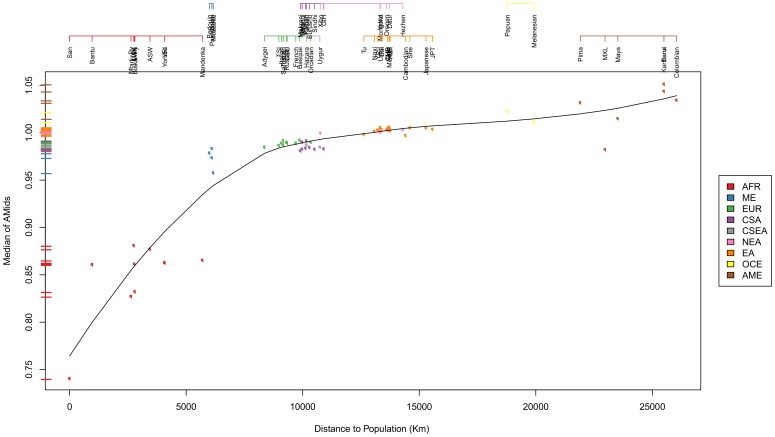
Genetic vs. geographical distance to the San coordinate. The median of the San AMids for each population is plotted against the geographical distance to the San. With increasing geographical distances we observe an increasing gradient of genetic distances. The distance is the smallest for the other two Hunter Gatherers and still close to the other African populations, decreasing substantially for ME. It further decreases for CSA/Europe and EA. The Oceanic and American populations have the largest distances to the San. The two most distant populations are Karitiana and Surui, both from South America.

Africa has two sub-blocks: the hunter gatherers (San, Biaka Pygmies, and Mbuti Pygmies) and agriculturalists (Bantu, Mandenka, Yoruba, Luhya, Masai and African-Americans). The distance to the six African references provides a global view of human migration and the out-of-Africa expansion of modern humans [14]–[15]. The distance between the HG and the non-African populations is larger than the distance of agriculturalists to the same populations and we can infer that the ancestors of non-Africans in the out-of-Africa human expansion were closer to the current agriculturalists than to the HG [9] [16]–[17]. We investigated the correlation between the geographic and the genetic distance (see Fig. 6 and Table S2) to the Bantu reference. The Africans are present at one end of the gradient with the American and Oceanic populations, with the lowest African coordinates, located at the opposite extreme.

The populations from NA, ME, Europe and CSA form a large block with 3 sub-blocks (NA/ME, Europe, and CSA). The Mozabites, the single North African population in HGDP, are closer to the Middle East than to the sub-Saharan populations. Many Europeans have several high AMids showing their low degree of isolation. CSA individuals have the lowest AMids to their own references, reflecting high gene flow during the last 80,000 years, with populations migrating and settling from many directions and contributing to numerous expansions [18] (this is best observed in the non-normalized AMids – Fig. S3).

The East Asians show several high AMids with a South/North Asia pattern, which is gradual and continuous, suggesting gene flow between these populations. Although not geographically close neighbors, the Hazaran and Uygur are very similar to one another, as previously reported [19]–[20]. These two populations show strong similarities to both CSA and EA, although closer to EA references. The most similar references to the Papuans and Melanesians are from South East Asia, consistent with the expansion of those populations to populate Oceania circa 50,000 years ago [21]. Similarly, the closest references to the five American populations are from northern East Asia as would be expected from the migration of individuals across the Bering strait to colonize the Americas around 20,000 years ago [22].

Isolated populations can be identified in Fig. 2 as they show high AMids for their own reference, with low values for neighbor populations. They include the San, Mbuti and Biaka Pygmies, Kalash, Pima, Colombian, Karatiana, Surui, Melanesian, and Papuan.

### Sub-Saharan African Block

**Figure 7:**
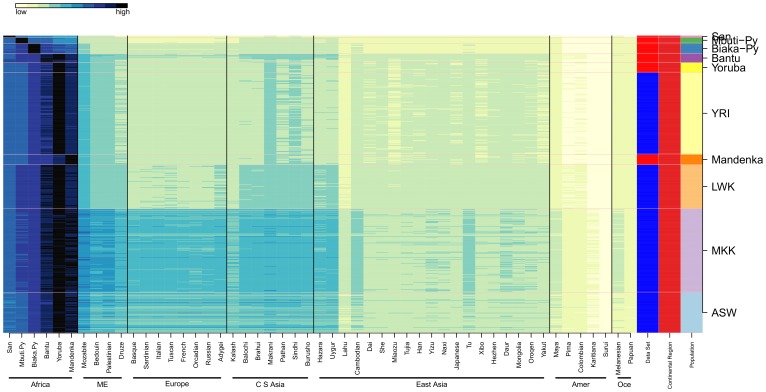
AMids of Sub-Saharan Africa. There is separation between HG and Agriculturalists. The Biaka are closer to the Agriculturalists than the Mbuti or San, reflecting the gene flow between Biaka and the neighboring Bantu. The AMids for HG to their own references are quite high and distant from their second highest AMid, indicating isolation. We don’t observe the same for the agriculturalists. Many Bantu have high AMids for the agriculturalists, especially Bantu and Yoruba and a wide range of individual variation, representing the heterogeneity of the Bantu HGDP population, and possibly reflecting that the Bantu were collected at seven locations. The Yorubas (mainly from Kenya) have a high Yoruban AMid but also strong Mandenka and Bantu. The two Yoruba populations (YRI/HapMap and Yoruba/HGDP) are indistinguishable. The Mandenka (mainly from Senegal) show a strong Mandenka AMId and are the most isolated of the agriculturalists. Africans have the highest non Sub-Saharan AMids for Mozabite (North Africa), Bedouin (Middle East) and Makrani (CSA). The Mozabite and the Bedouins are quite close geographically, while the relative closeness of the Makrani might reflect the history of slavery in Balochistan, Pakistan.

AMids of individuals of African origin show that HG differ from the other African populations (see Fig. 7) as previously described by Jakobsson et al [23]. The San are the most isolated and the two Pygmy populations are close to one another but differ in their relationships with other African populations, which reflects their population histories as the Biaka Pygmies (from Central Africa) have mixed with nearby Bantu and Sudan villagers [18] and the Mbuti Pygmies (from the Ituri Forest in Zare) are more isolated; they diverged around 19 to 26 thousand years ago [24].

The agriculturalists are similar to each other, with the Bantu the closest to the HG. The gene flow between agriculturalists and HG is the highest between Bantu and Biaka Pygmies as suggested by the reciprocal genetic similarity.

The African Americans from the Southwest of the US (ASW) and the Masai, from Kenya (MKK) show the highest ME/European/CSA AMids. For MKK this could be explained by a Neolithic farming migration from ME to Africa, or a more recent Arab migration [25]. For ASW, this could be explained by the admixture that occurred in the US between slaves from Africa and individuals of European ancestry [9]. The non-African AMids contain information, e.g., the ASW are further distinguished from the MKK because of differences in ME/European/CSA AMids. This distinction is not as clear when only African AMids are used for comparison; the greatest depth of information is provided by considering all 51 AMids, not only the highest ones.

The closest populations to the sub-Saharan block are the Mozabite, Palestinian, Bedouin and the Makrani. The first three populations are geographically close to sub-Saharan Africa, while the similarity with the Makrani, from Balochistan, Pakistan, may be explained by historical factors, supported by genetic studies, because the Makrani are the descendants from slaves brought to Pakistan by the Arabs, especially from areas in present day Mozambique [26]–[27].

The Yoruba from HapMap and from HGDP show indistinguishable AMids, indicating that both datasets can be analyzed together.

AM analysis of HGDP and HapMap provides higher resolution than previously published using Frappe and PCA [28] [20] [19]. In AM, for instance, individuals from the different sub-Saharan Africa populations can easily be distinguished amongst themselves or compared with all the other populations, while using Frappe and Structure, they are indistinguishable and clumped into a single cluster in the global analysis [28] [20]. PCA provides some level of distinction [19], although not as detailed as what is observed in the current analysis (see Fig. 7).

### North-Africa, Middle East Block

North Africa and the Middle East are represented in HGDP by the Mozabite, Bedouin, Druze, and Palestinians (Fig. S4). The only North Africans are the Mozabite, collected in Algeria. Our global analysis suggests that the Mozabite are a transition population, the closest to sub-Saharan Africa than any other, but with stronger similarity to Middle East and Europe (see also [29]). The Mozabites are the most isolated of this block, as already mentioned in [30]. This could also be due to a bias, reflecting a weakness of sampling of this part of the world from HGDP, with direct impact on Ancestry Mapper. The Bedouins show high heterogeneity, and are less similar to Europeans than Druze and Palestinians are. The Druze and the Palestinians show similarity with one another, with high western European coordinates. The two populations have historical connections and there are Christian Palestinians of Druze origin. We do not see the Druze isolation mentioned by Shlush et al [31]. The Makrani population is relatively close to the Druze and Palestinians.

### European Block

The genetic distance to Africa is larger in Europeans than in CSA or ME individuals, as reflected by smaller African AMids for Europeans (Fig. 2). This finding could be explained by Europeans having double ancestry, none directly from Africa: i) Neolith revolution and expansion of farming 10,000 years ago [32]–represented here by the Palestinians and the Druze, and ii) CSA migration to Europe 35,000 years ago [33]–[34].

Strong gene flow within Europe is indicated by the several high European AMids for Europeans, with the only exception being the Adygean. Each population can be distinguished not only by its highest AMid, but by the overall “barcode”, provided by the full set of AMids (see Fig. S5).

The Basques, the Adygean and the Sardinians show the highest isolation of European populations (see also [35]–[36]). Basque and Sardinians have the lowest AMIds for CSA, raising the possibility that they were less influenced by the human migration from CSA. There is a discernible Asian reference in both Russian and Adygey. The Russians are closer to the Europeans than to the Asians, which may reflect that the HGDP Russian collection was done at St. Petersburg, in the western part of Russia. The Adygey, from the Northern Caucasus, are the most distant to all other Europeans and are not particularly close to the Russians either.

Of the three Italian populations, the Sardinians show differences in many AMids from Italians and Tuscan, which are closer to one another. The Tuscan from the HGDP are indistinguishable from the Tuscan from the HapMap (TSI).

### Central South Asia Block

Many populations in the CSA block show the smallest AMids to their own references and to any other references (see Fig. S3, showing the non-normalized AMids). This might reflect the great gene flow present in CSA, reflecting inward and outward populations expansions [18] [37].

In contrast, the Kalash, from Northern Pakistan, have high AMids for their own reference and low for others, indicating that they are one of the most isolated groups (see Fig. S6). The Kalash are geographically and culturally isolated and they are animists while their neighbors are Muslims [38]. In AM they are almost as distant from Europe than to CSA (also discussed in [39]). The Burusho, a linguistic isolate show isolation, which previous studies have not reported [26].

Distinction between populations can be done by the level of EA AMids: low for the Kalash, medium for the Balochi/Brahui/Makrani, high for Pathan/Sindhi and the highest for the Burusho and GIH.

Some similarity between Pathan and Sindhi has been reported [28] [20] and our data shows they have similar barcodes, with many high AMids. Some Pathan have the highest European AMids of CSA, which could be due to some level of Greek ancestry, previously suggested to have originated from soldiers of Alexander the Great [40].

The highest AMid for the Gujarat Indian HapMap (GIH) is Pathan, the closest geographically population to Gujarat. The African AMids of GIH individuals are the highest of all non-African populations. This is consistent with the delayed expansion hypothesis, in which the GIH are the descendants of an ancestral Eurasian founding population, isolated long after the out-of-Africa diaspora, before expanding throughout Eurasia [37]. Including references from Indian origin in AM would further elucidate this topic; the absence of Indian populations in HGDP, is a limitation noted from the onset of the project [7].

### East Asia Block

The East Asian block is composed of 18 populations, the highest number of any block. The populations in EA are similar to each other with several high AMids (see Fig. S7). Disregarding the pair Hazara/Uygur, five sub-groups are distinguishable and differentiate among a South-North gradient [41]. The sub-group Tujia/Han/Yizu/Naxi are geographically from central China and experienced gene flow from all their neighbors, reflected in a barcode with high EA AMids for both South and North. The Han have high AMids for Dai, She, Miaozu, Tujia, Han, Yizu, Naxi, Japanese, Tu, Xibo and Mongolian. The observed diversity of the Han makes sense in historical terms; Han are the biggest ethnicity in the world, comprising 20% of the world population (1,300 million people) and have mixed with their neighbors extensively. The three different Han datasets (two from HapMap and one from HGDP) show great similarity, with the Dai AMid higher in several individuals from the HapMap collection of CHD (Chinese Han, collected in Denver, US). The two Japanese populations (one HGDP, other HapMap) are indistinguishable. Hazara and Uygur have a similar barcode, with EA AMids as the highest, but with higher CSA AMids than any other EA population; they represent transitional populations between CSA and EA. We analyze Hazara in the context of the EA block, contrary to previous studies which placed it in CSA [28] [20].

The Yakut from Sakha, Yakutia, an autonomous republic of Russia, are shown to be strong isolates. The Cambodian, the Lahu and the Japanese are relative isolates.

### Native American Block

The population from the Americas have the highest AMids for Native-American references (see Fig. S8). The Maya have high EA AMids which is expected as the individuals that populated the Americas came from the North of East Asia, crossing the Bering Land Bridge, around 17,000 years ago [22] [42]. The Maya are closer to East Asia and Europe and are the least isolated. The Pima and Maya in the North are genetically closer to East Asia than Surui, Karitiana and Colombians in the South. Curiously the Pima, the most northern population show less similarity with EA than the Maya does. The highest EA AMids in the Native Americans correspond to those references closest to the Bering strait, namely Mongolia, Henzhen, Daur and Oroquen. The Native Americans are genetically the most distant from the Africans, reflecting the longest journey coming from Africa and the accumulation of successive founding effects.

AM identifies the Mexican from HapMap (MXL) as the most heterogeneous, with varying barcodes in the European and American AMids. This reflects the mixed origin of Mexicans, who have a history of admixture between Europeans and native Americans (symbolized by Martin, the first Mestizo, the son of Malinche, a Nahuan and Hernán Cortés, the European conqueror of Mexico). Some Mexicans have the highest African AMids of Americans, likely due to admixture introduced through African slaves, brought to Mexico in the early part of XVIII century. Ancestry Mapper is able to correctly highlight this three-way admixture in Latino populations, which previous work has noted as particularly challenging [43].

### Oceanic Block

The Oceanic block contains the Papuan and Melanesian, both of which are shown as isolated (see Fig. S9). The largest genetic distance of Oceanic individuals is to sub-Sahara Africa, which is expected, as Oceanic populations are at the end of the out-of-Africa journey. The second largest distance is to the Native-Americans, an intriguing observation, as both Oceanic and American populations descend from East Asia (albeit with a South/North difference in origin). This may be explained by different migration date periods: humans settled in Oceania 50,000 to 30,000 years ago [21], many thousands of years before they settled the Americas. Also, it could be due to a double founder effect: the small number of settlers of the Americas (from the North) and Oceania (from the South) resulting in high genetic differences. The highest EA AMids in Melanesians are Cambodian, Dai, Han and Yizu, from South of East Asia, contrasting with the Northern origin of the highest EA AMids for the Maya, again reflecting the different geographical ancestry of the two continents.

### Clustering AMids

Clustering ancestry data highlights relationships between individuals: a) individuals of different populations clustering together, indicate similarity of populations, b) individuals of the same population not clustering together, indicate heterogeneity in the population, and c) clusters exclusively of individuals from a single population, indicate a genetic isolate.

We clustered AMids with PAM, [13] but other algorithms can be used. We used a fixed number of clusters (K) from 2 to 40. Small Ks define continental groupings corresponding to initial migration events, while increasing K correlate to regional events, sometimes showing isolated populations in their own clusters. The population composition of the clusters and how they evolve with increasing number of clusters mimics time and roughly corresponds to human migrations (see Table 1). An animated figure with the population distribution in clusters with increasing K is shown in Supplementary.

**Table 1:** Description of HGDP/HapMap clustering of AMids, from K = 2 to K = 40.

K	Events	Notes
2	Africa vs. non-Africa	Three ASW placed in non-African cluster
3	IndoEuropean vs. EA/AME	MXL and Papuan with IndoEuropeans; Melanesians w Asians
4	MKK/ASW vs. rest of Africa	Papuans with MKK/ASW cluster
5	ME/Europeans vs. CSA	Kalash/Balochi/Brahui/Makrani split between European/CSA cluster; MXL, Melanesian w CSA
6	AME vs. EA	Most MXL w CSA cluster, some in AME Cl
7	Papuan and Melanesian	All Melanesian and Papuan in their own cluster
8	Hazara/Uygur vs. CSA	MXL with Hazara/Uygur
9	Mozabite/Bedouin vs. Europe/ME	Palestinian, Druze in both
10	HG vs. other Africans	San and both Pygmies split fr other African Populations
11	Hazara/Uygur vs. MXL	Most MXL in its cluster; few in European, others in CSA
12	LWK vs. other Africans	Some Bantu w LWK
13	CSA in 2 Clusters	Kalash/Balochi/Brahui/Makrani vs. Pathan/Burusho/GIH; Sindhi in both
14	ME/South Europe vs. Western Europe	Cluster with Mozabite/Bedouin (ME), Basque/It/Tuscan (Europe) vs. other ME, other European
15	Native America split	Karitiana/Surui vs. Maya/Pima; Colombian in both
16	MKK/ASW split	
17	Yakut vs. EA	Yakut in own Cl; 1st population-only cluster; few Mongolia/Oroquen in Yakut Cl
18	Melanesian vs. Papuan	One Papuan in Melanesian Cl
19	Palestinian vs. S Europe	Some Mozabite/Bedouin w Palest; most Druze w Palest
20	Balochi heavy vs. ind fr many pop	Balochi-heavy vs. Bed/Pal/Druze/Kal/Balochi/Brahui/Mak/Pathan/Sindhi
21	MKK/ASW split	Most MKK in one of the clusters; ASW split between three clusters
22	Mozabite vs. Bedouin	Mozabite, Bedouin mostly in their own cluster
23	Mexican into 2 clusters	One cl with some Mayan individuals
24	Biaka-Py vs. Mbuti-Py/San	
25	Karitiana vs. Surui	
26	Russian/Adygey vs. Western Europe	
27	Basque/Sardinian vs. Europe	Most Basque/Sardinians in one Cl
28	South vs. North Asia Cambodian/CHB/JPT split	
29	Maya vs. Pima	
30	Kalash vs. CSA	
31	MXL split	MXL in 3 clusters; one with few Maya
32	Bantu/Yoruba/YRI/Mandenka divide	Individuals of those 4 populations form two clusters
33	Burusho vs. GIH	
34	One MKK-heavy Cl split	MKK individuals now in 2 main Cl
35	Balochi heavy; individuals many pop	Rearranges more than 1 Cl: GIH/Burusho joined single cluster; Balochi/Brahui/Makrani/Pathan/Sindhi
36	South vs. Central East Asia	Lahu/Cambodian/Dai vs. Han/CHB/CHD; split: She/Miaozu/Tujia/Yu; JPT now in NEA Cl
37	It/Tuscan/TSI vs. West Europe	Italians/tuscans in one cluster; CEU/French/Orcadian in two clusters
38	Japanese vs. North Asia	Japanese/JPT vs. Tu/Xibo/Daur/Mongolian; split: Hezhen/Oroqen
39	Mbuti vs. Biaka Pygmies	All individuals of Biaka, Mbuti, San are now in their own cluster
40	ASW split	ASW now in 5 clusters; one has the majority of them; some have ASW/MKK

Clustering HGDP and HapMap.

**Figure 8:**
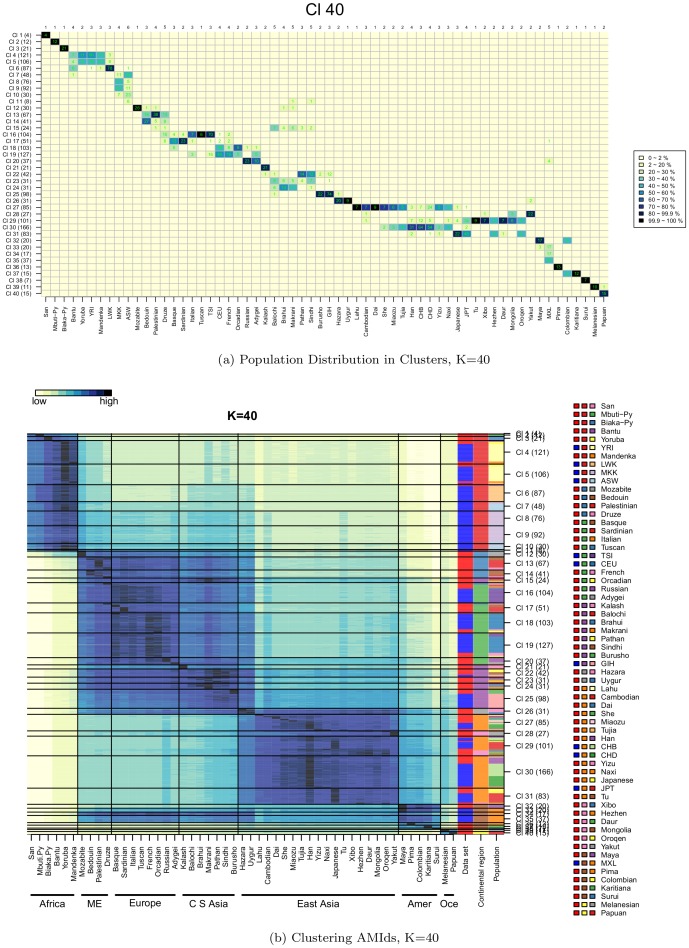
Clustering of HGDP and HapMap individuals, K = 40. (8a) Distribution of individuals of each population, per cluster. (8b) AMIds for each cluster. Continental regions are in adjacent clusters. For some populations all individuals are in a single cluster (San, Mbuti and Biaka Pygmies, Mozabite, Tuscan, Uygur, Lahur, Dai, Karitiana, Surui and Melanesian). Some clusters are composed of only a single population. Population heterogeneity is indicated by individuals in several clusters (e.g., ASW, Makrani, Sindhi, Druze, and MXL). Similarity between populations is shown by clusters with more than one population (e.g., cluster 16 with Tuscans/HGDP, TSI/HapMap and Italians). Mozabite are in a single cluster, with strong African and ME AMids, suggesting they are a transition population. ME individuals are mostly placed into 2 clusters (Bedouin vs. Palestinian/Druze, which has higher European AMids). Western Europeans are in three clusters with slightly different European AMids. The Eastern Europeans, Russian and Adygey, are in the same cluster. Balochi and Brahui/Makrani are in two clusters. The GIH co-cluster with the Burusho, with strong AMids for Burusho and Pathan. Hazaran and Uygur are in a distinctive cluster with strong AMids for both. South East Asians are mostly in a single cluster (Lahu, Cambodian, and Dai, with some She, Miaozu and Tujia). A Northern East Asia cluster has most individuals from Tu, Xibo, Daur and Mongolia, and its highest AMids correspond to those populations. All three Han populations are mostly in a single cluster; so are the two Japanese. Most East Asian populations fall in more than one cluster, except for the Yakut, comprising a single cluster and showing their isolation. Four of the Native American (Maya, Pima, Karitiana and Surui) produce their own clusters; the Colombians split between the Karitiana and the Maya cluster. The Oceanic populations are split into two clusters (one with all Melanesian and a Papuan; the other with only Papuan). In the Supplementary we present an animated figure with the distribution of individuals from K = 2 to K = 40.

For K = 2 we observe the big genetic difference of Africa vs. the rest of the world: all individuals of African origin (except for 3 ASW) are in one cluster and all non-African individuals in the other. K = 7 separates individuals into broad continental groups. The African are in two clusters (MKK/ASW vs. the remaining African). ME and European are in a single cluster; as are the Pakistani/Indian populations; individuals from CSA populations are split between these two clusters. Individuals of East Asia, of Americas and Oceania are each in their own cluster. For K = 40 we observe a division of the continental groups into several close clusters (see Fig. 8). The African HG and Luhya are in their own clusters. The Masai and ASW are in similar clusters, with a gradient of ME and European AMids differentiating between them. The Mozabite are placed in the transition between Africa and ME and in their own cluster. ME separates into several clusters, distinctive in the relevance of the European AMids. The Europeans are in four clusters: a Basque/Sardinian; Italian; Western Europe; and Eastern Europe. The Kalash are placed in a distinctive cluster. The CSA is divided into two main groups: one with Balochi/Brahui/Makrani, the other with Burusho and GIH in a single cluster. One cluster is composed of all the Uygur and all but one Hazaran showing them as genetically similar and between the CSA and EA regions. East Asia divides into South, North, Han and Japanese clusters. The Native Americans are placed into their own clusters, with the Colombian split into the Mayan and Karitiana clusters. The populations from Oceania make up two clusters, one with all Melanesians and one Papuan; the other with only Papuan.

**Figure 9:**
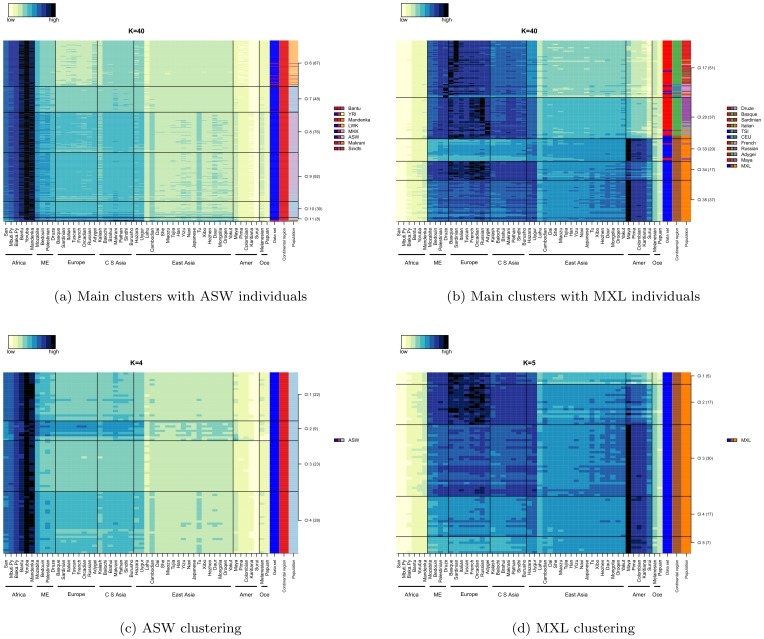
Clusters with ASW and MXL individuals. (9a) Clustering of HapMap and HGDP, K = 40, showing only clusters with ASW. ASW individuals are present in mostly three clusters, with most visible differentiation in the European AMids. The smallest of the clusters has six ASW, with much higher European AMids. One Sindhi and one Makrani, members of this cluster, are the same two individuals highlighted by Lopez Herraez et al ([19] and personal communication from Mark Stoneking); we agree with the authors that they are of recent African admixture. (9c) Clustering exclusively ASW individuals (K = 4) shows the separation based on European AMids, with cluster 2 showing the highest AMids for Europeans. (9b) Clustering of HapMap and HGDP, K = 40, showing only clusters with MXL. Mexicans are present mostly in three clusters, with the distinctions in the European and Native-American AMids. Mexicans in clusters 34 and 35 have higher African AMIds than Europeans or Maya, suggesting a level of African admixture for some Mexicans. (9d) Clustering exclusively MXL (K = 5) shows the separation based on European and American AMids.

The ASW are African Americans living in the Southwest of the US. For K = 2 three ASW are placed in the non-African cluster, the only individuals with African origin in that cluster. For K = 40, ASW fall into 6 clusters, each with different levels of African and European AMids, indicating individuals with different degrees of admixture (see Fig. 9a). In many clusters ASW is associated with MKK. Interestingly, based on F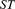, the HapMap consortium places ASW closer to YRI [10], but on close examination their own PCA shows ASW closer to MKK, similar to our clustering results. Clustering only ASW produces clusters with differentiated levels of African/European AMids (see Fig. 9c). One ASW cluster has the strongest European AMids of all individuals of African origin and include the individuals that co-cluster with non-Africans for K = 2.

MXL is composed of Mexicans, collected in LA, US. At K = 40, MXL individuals are mostly present in three clusters (see Fig. 9b), showing their heterogeneity, with different barcodes, differing in the proportion of American and European AMids (high European AMids; high American AMids; and the third cluster is mixed).

### Ancestry Assignment Using AMids

**Figure 10:**
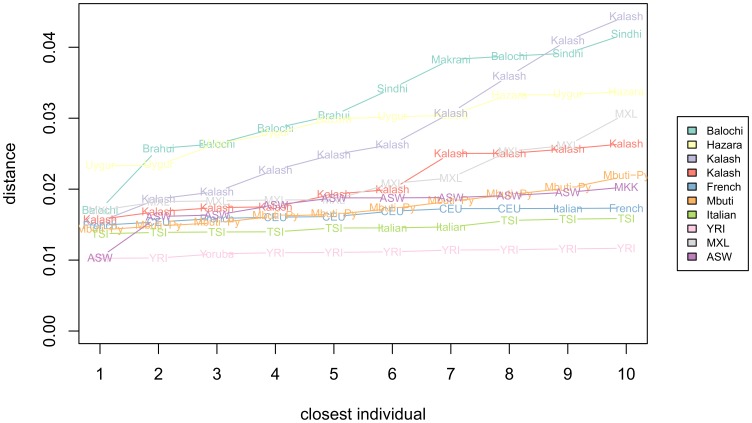
Ancestry of closest individuals based on distances of AMids. For 10 individuals we calculated the Euclidean distances of barcodes of AMids to all the individuals in our dataset, and show the ancestry of the ten closest. Some individuals are closest to individuals of only the same ancestry (e.g., the MXL and the Mbuti-Py), while others are closest to individuals of different origins (e.g, the French is closest to 6 CEU, 3 French and 1 Italian).

AMids can be used to assign ancestries, in a “guilt by association” approach, comparing the AMids of an individual to the AMids of individuals of known ancestry. To investigate the use of AMids in ancestry assignment we selected ten individuals and calculated the Euclidean distances of the AMids to all individuals in HGDP/HapMap. The ancestry of the closest individuals is indeed a clear indication of ancestry (see Fig. 10 and Table S3). For some individuals all ten closest ancestries are the same (MXL, Mbuti-Py, Kalash and Yoruba). Others are closest to individuals of different populations, e.g, the French are close to six CEU, three French and one Italian. The Hazara individual is closest to six Uygur and four Hazara, consistent with the similarity of the two populations we observed throughout this work.

## Discussion

We present Ancestry Mapper, which gives individuals an Ancestry Mapper Id (AMid), providing world-wide representation, by calculating the Euclidean distance to references of HGDP. The advantages of AMids include: a small, stable and fixed vector of 51 numbers which can be used in data mining and clustering algorithms; intrinsic ancestry information; local and global information in the same analysis; dataset context-independence; and dataset independence (when there are enough common SNPs of high quality with the HGDP references). Ancestry Mapper can provide greater detail than other methods, such as Frappe, or characterization by Principal Components. For instance, in the context of a single global analysis of HGDP, Ancestry Mapper distinguishes clearly between hunter gatherer and agriculturalist African populations while analysis performed by other methods result in less discrimination and often require secondary analysis.

AMids provide genetic distances to close and distant populations, e.g., a Mongolian is compared to a neighbor population such as the Daur, but also to the geographically and genetically distant African Yoruba population. Admixtures are easily observed, e.g., an individual with both African and Asian ancestry will have high AMids in both Asian and African references.

Ancestry Mapper analysis of the HGDP and HapMap produces distinct blocks (Africa, NA/ME/EUR/CSA, EA, Oceania, and the Americas), reflecting human history, highlighting the relationships between populations and recapitulating human migrations. We differentiate between the different populations of the African block, with a clear distinction between HG and agriculturalists. The block NA/ME/EUR/CSA shows that the Indo-European populations are similar to each other, providing genetic evidence to the long history of migrations and confluences within this region. Population-specific characteristics and regional relationships are observed, e.g., individuals from ME show intra-regional diversity and similarity to Africa and Europe. In Europe we observe similarity between geographically close populations (France, Italy and Tuscany), while others are confirmed to be isolates (Basques and Sardinians). The Russians have strong eastern Asia coordinates. Central South Asia has the highest overall similarity to all other blocks (Europe, East Asia, but also relatively strong to Africa), validating that CSA is positioned as a confluence of migrations from many different parts of the world.

Ancestry Mapper genetic distances correlate well with geographical distances in Africa mimicking the out-of-Africa human migration (see Fig. 6). We observe a continuum South/North genetic gradient in East Asia populations. Ancestry Mapper allows to pinpoint a North-Eastern Asia influence in individuals from the Americas, which wanes in the most southern American populations, also correlating with geographical distance. The known events in human migration and expansion are also visible in the strong South-Eastern Asian influence in individuals from Oceania.

Clustering AMids recapitulates what is known about human migrations. The number of clusters correlates with migration time and historical events: for a small number of clusters we observe continental differences; more recent events, such as population isolates, appear for a larger number of clusters. Clusters are labelled by the highest AMids of its members with no need to include in the dataset individuals of known ancestry to serve as labels. For K = 40 some clusters are composed almost entirely of a single population, including the San, Mbuti and Biaka Pygmies, Mozabite, Kalash, Yakut, Maya, Pima, Surui, Melanesian, and Papuan, all known to be genetic isolates (one of the main reasons for their inclusion in the HGDP project [44]). Unknown isolates in any dataset could thus be identified, if they produce similar single own clusters. Individuals from the Mexican population of HapMap are present in mostly three clusters, with some individuals showing strong European and/or American AMids, which is explained because of recent admixture (XVI century) between Native Americans, Europeans, and Africans.

Assigning ancestry can be done by calculating distances of Ancestry Ids and relating an individual of unknown origin to the closest individuals for which ancestry information is available.

Because Ancestry Mapper is based on HGDP it suffers from its limitations. For instance there is less coverage of India and deeper coverage in China and Pakistan [45]. Some regions of the world do not have enough populations, such as the North of Africa, represented only by the Mozabites. This limitation is mitigated because Ancestry Mapper does not rely solely on the highest AMid but on the information available in the barcode of the 51 AMids – the medium and lower references are also informative. The power of AM would be increased by including more references, ensuring a more detailed coverage. With more AMids, Ancestry Mapper would better differentiate ancestry in individuals. Information on how to expand the reference set is included on the Ancestry Mapper R package. We plan in our future work to improve AM by substantially increasing the number of references. Ancestry Mapper will be easily expanded to exome or whole-genome data by using individuals publicly available as references and we are currently working on it.

We believe Ancestry Mapper provides a powerful simultaneous global and local approach to human diversity, by placing every individual in a world-wide genomic map. We see AM as a powerful tool in ancestry assignment, analysis of human expansion and migrations, and in the study of genetic diseases where stratification is important.

## Materials and Methods

### 0.1 Data Sets

We used two datasets: HGDP and HapMap. The Human Genome Diversity Project is a collection of 51 populations, 938 individuals, with world-wide coverage [7]. We obtained the data set at http://hagsc.org/hgdp/files.html. Population size ranges from 5 to 46 individuals, with mean size of 17. The data set comprises 644,285 autosomal SNPs. HapMap is an international effort to cover human diversity [10]. The data set comprises 1,654,989 SNPs for 1,184 individuals, of 11 populations; population size ranges from 50 to 144, with mean size 89. We obtained the HapMap data set at http://hapmap.ncbi.nlm.nih.gov.

### 0.2 SNP Selection

We used plink [46] to merge the HGDP and HapMap datasets, and selected SNPs with missing call inferior to 1% (geno option to 0.01), which yielded 289,160 SNPs.

### 0.3 Individual Characterization

Each SNP has two alleles, coded 1 or 2. To the subsequent analysis we need a single number per SNP, which we obtain by adding the two genotypes. Possible combinations are 2 (both genotypes coded 1), 3 (cases of 1 2 and 2 1) and 4 (both genotypes 2). This makes for a smaller data set and provides the same code for the heterozygous SNPs (1 2 and 2 1 will be the same). In this way, every individual is characterized by a vector of integers, easily comparable.

### 0.4 Reference Selection

To obtain the individual which will be used as reference for each population we 1) calculate the Euclidean distance between all individuals in each population, and 2) select the individual with the smallest median distances to all other individuals.

### 0.5 Hierarchical Clustering of References

We calculate the distances amongst the 51 individuals selected as references. We produce a clustering plot using the hclust function in R.

### 0.6 Calculation of 51-AMids

We calculate the Euclidean distance, normalized by the number of SNPs, between each individual and the 51-HGDP-based references. This process is independently done for each reference. Also each individual is independently calculated, as a stand-alone process, regardless of its data set. We can obtain AMids for a single individual coming from any dataset provided we have a reasonable overlap of SNPs with the 51 individuals used as references.

We normalize AMids by making the highest AMids 100, the lowest 0, and adjusting all others accordingly. Normalized AMids place the individual in the genomic map, forcing it to be committed to one reference, even if the absolute similarities, as indicated by the Euclidean distances, are not very big. They provide a global overview on the number of relevant references for each individual. The R Package Ancestry Mapper is available to produce AMIds.

### 0.7 Calculating Geographical Distances

We used the site http://www.movable-type.co.uk/scripts/latlong.html to calculate the geographical distances. We used the distances in straight lines, always by land. For instance the distance of Bantu to the Pima was obtained by adding three different steps. The median distances of every population to the San references were calculated and plotted against the geographical distances. A local correlation line was calculated using “loess”, an R local polynomial regression fitting function.

### 0.8 Visualization

We produce plots to visualize AMids, which are color coded depending on the value. Also shown is the individual’s origin. The plots use the function “image” in R and the color code from Color Brewer 2.0 (http://colorbrewer2.org/), via the RColorBrewer R package. The R Package Ancestry Mapper is available to visualize AMIds.

### 0.9 Clustering

We cluster AMids using the clustering algorithm Partitioning Around Medoids (PAM) [13] with the Euclidean distance as metric. Other clustering algorithms could be used, with the AMids as input data. PAM is available in the R package “cluster”, is non-parametric, relatively robust to outliers and takes a dissimilarity matrix. We start the clustering with K = 2 and analyze the results obtained when increasing the number of clusters until K = 40.

### 0.10 Ancestry Assignment

We calculated the Euclidean distances between AMids of selected individuals and all HapMap/HGDP individuals. We sorted the individuals by the smallest distance, indicating proximity and analyzed the ancestry of the ten closest individuals.

### 0.11 Software

Plink was used [46] to extract and get all SNPs in the same strand, produce genotypes with 1/2 codes and for quality assessment of SNP data. The R statistical language and environment [47] was used in most of the analysis, including the visualization, plotting data and clustering algorithms. Python was used to parse data and in some of the analysis.

### 0.12 R Package

The R Package Ancestry Mapper with functions to analyze data sets using the 51-coordinate system is available at CRAN: http://cran.r-project.org/.

## Supporting Information

Figure S1 **Hierarchical cluster of HGDP references.** The hierarchical clustering of the references for Ancestry Mapper shows the African populations as one of two major branches of the tree. Within the African branch we observe the agriculturalists (Bantu/Mandenka/Yoruba) separated from the hunter gatherers (San and both Pygmies). The second branch contains two sub-branches: one is composed of American and Eastern Asia references, the other with the references from Central South Asia, Europe, Middle East and Oceania. The Oceanic are quite distant from the other references in its sub-branch. The branch containing the Indo-european references, is divided into CSA and Europe/Middle East. The Mozabite reference, from North Africa, is in a separate branch from the sub-Saharan populations, and closer to the Middle East.
(PDF)

Figure S2 **AMids calculated using for each individual a randomly selected set of 20,000 SNPs.** We calculated each AMid with a different, randomly selected set of 20,000 SNPs. The overall results are quite similar to the results using the original set of over 200,000 SNPs shown in Fig. 2.
(PDF)

Figure S3 **Non-normalized AMids of all HGDP and HapMap individuals.** We show for every HapMap and HGPD individual the Euclidean distances to all 51 references. This non-normalized AMids and the normalized AMIds shown in Fig. 2 are similar and in some cases compliment each other. For example, the individuals in the Surui population show very high normalized AMids for their own Surui reference, obscuring the relationship with other populations. Yet, from the non-normalized AMids we observe they are the most similar to the Maya, Karitiana, and Colombian. Also, the relative low intensity of the CSA individuals to their own references, is suggestive of less isolation than for other populations, information less obvious in the normalized AMIds.
(PDF)

Figure S4 **AMids of Middle East and North Africa.** The Mozabites from Algeria, North Africa are the most similar to all sub-Saharan Africa, even if they have large differences to the San and the Mbuti Pygmies and higher European than African AMIds. Mozabite are isolated as indicated by the high values for their own Mozabite reference, which could also reflect a bias in HGDP, as Mozabite are the only representative of North Africa and this negatively impacts Ancestry Mapper results for this region. The Bedouins are much less similar to Africa than the Mozabite. They have few high AMids, show similarity to European references and heterogeneity. Druze and Palestinians show several high AMids and are closer to the European references than to the Mozabite.
(PDF)

Figure S5 **AMids of Europe.** Many populations show several high European AMids, with Sardinians, Basques and Adygey the most isolated. The barcode makes each population unique. Russians and Adygey have strong East Asian AMids, which correlates with their geographical position between Europe and Asia. Italians are similar to Tuscans (both TSI/HapMap and Tuscans/HGDP) and it is difficult to distinguish between the two populations, with individuals showing high AMids for Italian, Sardinian and Tuscan. The third Italian population, from the isle of Sardinian, is clearly distinguishable and much more homogeneous, with high Sardinian and lower CSA/ME AMids. The HapMap CEU group shows heterogeneity, with Basque, French and Orcadian AMids producing distinctive barcodes that differentiates between individuals.
(PDF)

Figure S6 **AMids of Central South Asia.** Many populations show several high AMids, reflecting the many influences and migrations to and from CSA. The Kalash show isolation and are as similar to Europe as to CSA. The Makrani and the Balochi have a high level of intra-population heterogeneity. The EA references distinguish between the populations (e.g., Balochi/Brahui/Makrani have much lower EA AMids than Pathan/Sindhi/Burusho). Although Balochi, Makrani and Brahui live in the same region, Balochistan in Pakistan, differences can be identified. Brahui have stronger Brahui references. The Makrani have high levels of Brahui, Balochi and Makrani, and are heterogeneous with many individuals having different barcodes, but with the strongest influence being their own Makrani reference. The Balochi show even greater heterogeneity, with equally high coordinates for Balochi, Brahui and Makrani. Basque and Burusho languages have some similarity, but our data doesn’t show genetic similarity (supporting Ayub et all [48]). Two individuals (Makrani and Burushi) show high African AMids, possibly due to recent admixture, as previously reported [19].
(PDF)

Figure S7 **AMids of East Asia.** We distinguish several blocks according to the population barcodes, which follow a South/North gradient: Hazara/Uygur, Lahu/Cambodian/Dai/She/Miaozu, Tujia/Han/Yizu/Naxia, Japanese/Tu, Xibo/Mongolia/Hezhen/Daur/Oroqen, and Yakut. The Hazaran and Uygur show similarities to both CSA and EA. Hazara and Uygur are close to Northern EA AMids; Hazarian history mentions a link to the Mongolians, which has been confirmed in two genetic studies [27] [49]. We don’t see similarities of the Hazara with other Pakistanis, confirming previous studies [26]. The ancestry of Uygur is uncertain: some studies show a stronger European influence [50] and others a stronger EA influence [51]. Our results support the EA influence, although Uygur are closer to Europe than the other Eastern Asians, which might explain the conflicting reports. Several groups have several high AMids, especially the Northern populations. The three Han datasets are similar, as are the two Japanese. The Yakut and the Lahu are the most isolated. The Oroquen, although geographically the closest to the Yakut, show weak genetic similarity with them.
(PDF)

Figure S8 **AMids of the Americas.** The HapMap MXL population is more heteregeneous than the Native American populations. The Maya also has more high AMids than the other populations, indicating that they have the highest diversity and gene flow. The Surui population is quite isolated, being the most distant to many populations in the whole genomic map. Northern East Asia AMids are relatively high in Maya, probably reflecting the origin of the settlers of the American continent.
(PDF)

Figure S9 **AMids of Oceania.** The Papuan and the Melanesians are quite isolated, as shown by high AMids for their own references and low AMids for all others. Papuan and Melanesian AMids are high for the other Oceanic reference, with the lowest AMids for Africa. Melanesians have relatively high AMids for the South East Asia references (Cambodian, Dai, Naxi, Yizu and Han), probably reflecting the settlement of Oceania from individuals coming from South East Asia.
(PDF)

Table S1 AMIds for HGDP and HapMap individuals. Ancestry Mapper Ids for HGDP and HapMap individuals.
(XLS)

Table S2 Genetic and geographical distance to the Bantu. Genetic and geographical distance to the Bantu.
(XLS)

Table S3 Ancestry assignment distances between AMids For HGDP and HapMap individuals. Distances between AMIds For HGDP and HapMap individuals.
(XLS)

Movie S1 **Animated GIF Figure For HapMap/HGDP clustering, fast.**
(GIF)

Movie S2 **Animated GIF Figure For HapMap/HGDP clustering, slow.**
(GIF)
