# Supplementary figures and images for: HGDP and HapMap Analysis by Ancestry Mapper Reveals Local and Global Population Relationships
Source: PLoS One. 2012 Nov 26;7(11):e49438. doi: 10.1371/journal.pone.0049438 (PMC3506643; doi:10.1371/journal.pone.0049438)

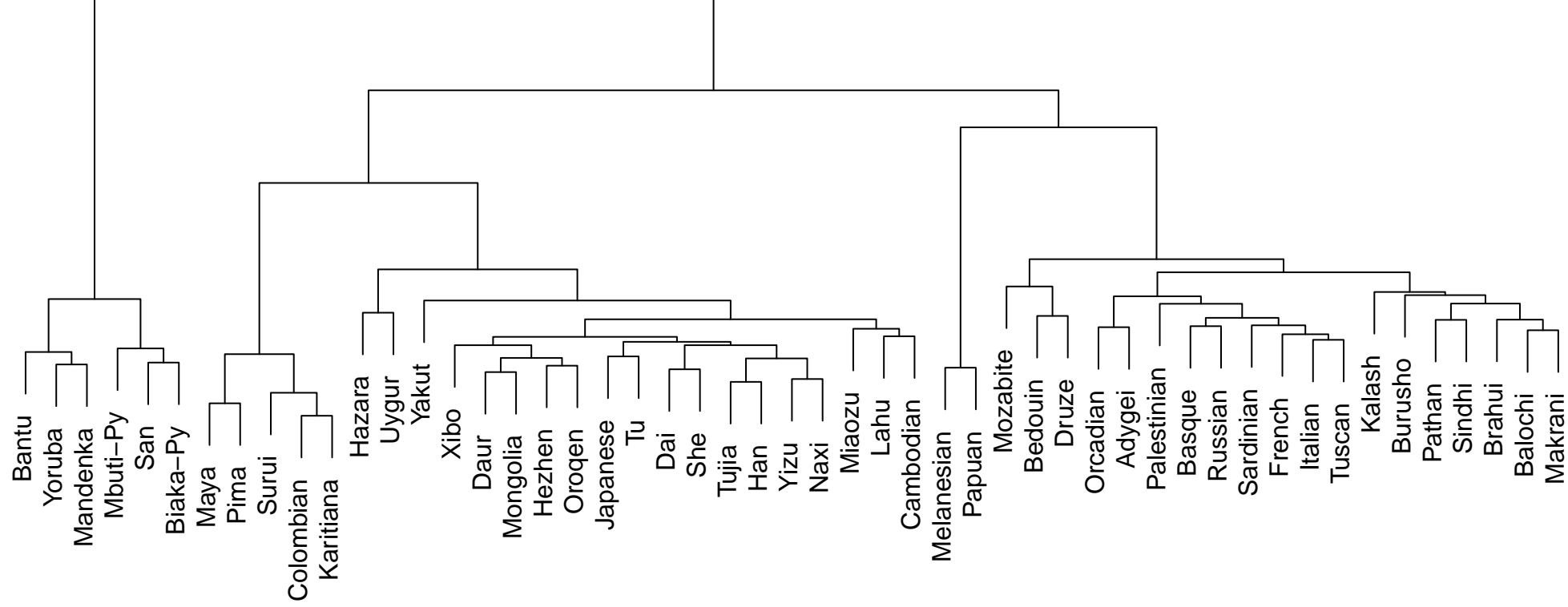

Supplement: Figure S1 — Hierarchical cluster of HGDP references. The hierarchical clustering of the references for Ancestry Mapper shows the African populations as one of two major branches of the tree. Within the African branch we observe the agriculturalists (Bantu/Mandenka/Yoruba) separated from the hunter gatherers (San and both Pygmies). The second branch contains two sub-branches: one is composed of American and Eastern Asia references, the other with the references from Central South Asia, Europe, Middle East and Oceania. The Oceanic are quite distant from the other references in its sub-branch. The branch containing the Indo-european references, is divided into CSA and Europe/Middle East. The Mozabite reference, from North Africa, is in a separate branch from the sub-Saharan populations, and closer to the Middle East. (PDF) [file pone.0049438.s001.pdf]

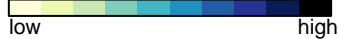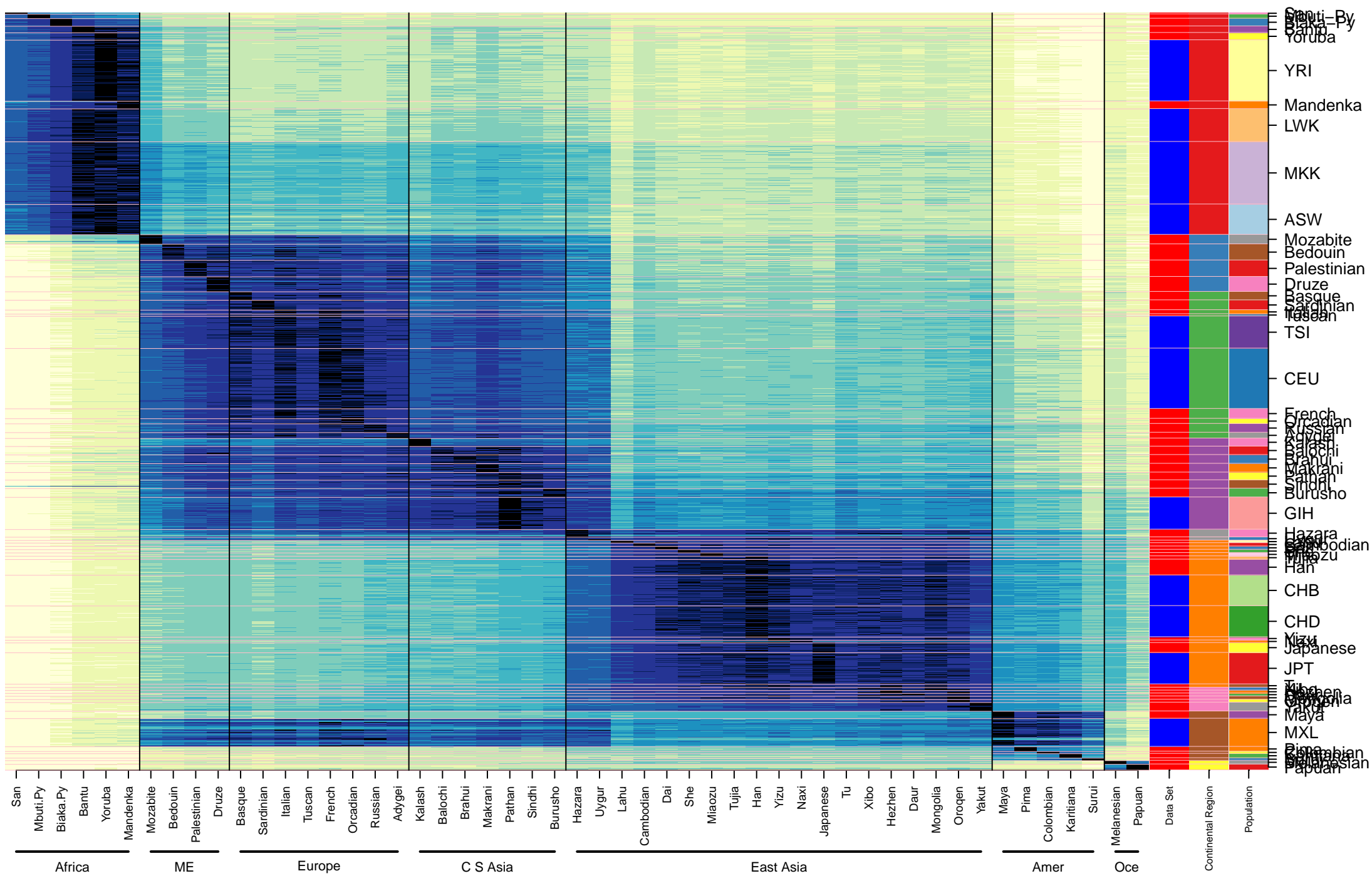

Supplement: Figure S2 — AMids calculated using for each individual a randomly selected set of 20,000 SNPs. We calculated each AMid with a different, randomly selected set of 20,000 SNPs. The overall results are quite similar to the results using the original set of over 200,000 SNPs shown in Fig. 2. (PDF) [file pone.0049438.s002.pdf]

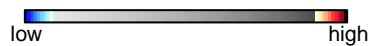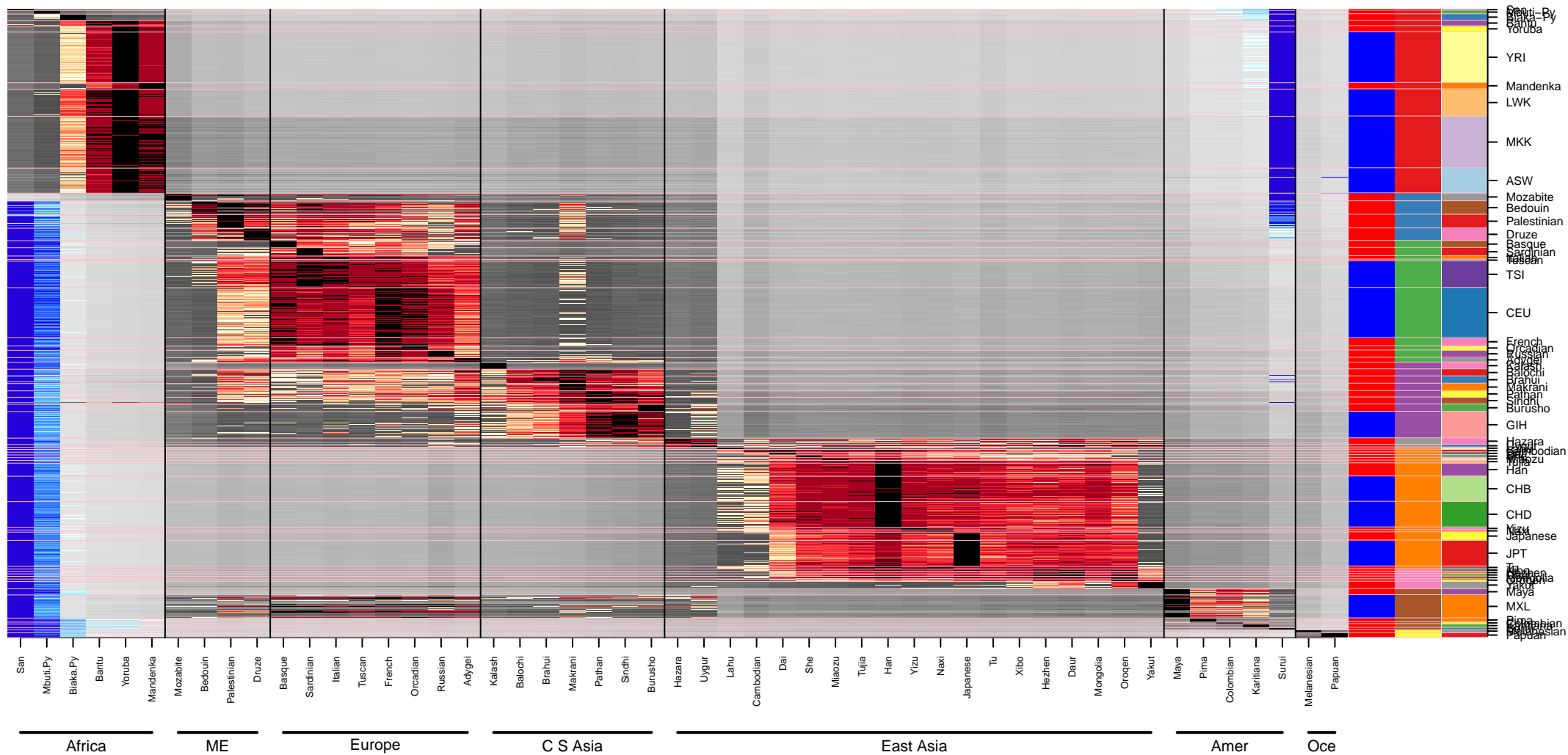

Supplement: Figure S3 — Non-normalized AMids of all HGDP and HapMap individuals. We show for every HapMap and HGPD individual the Euclidean distances to all 51 references. This non-normalized AMids and the normalized AMIds shown in Fig. 2 are similar and in some cases compliment each other. For example, the individuals in the Surui population show very high normalized AMids for their own Surui reference, obscuring the relationship with other populations. Yet, from the non-normalized AMids we observe they are the most similar to the Maya, Karitiana, and Colombian. Also, the relative low intensity of the CSA individuals to their own references, is suggestive of less isolation than for other populations, information less obvious in the normalized AMIds. (PDF) [file pone.0049438.s003.pdf]

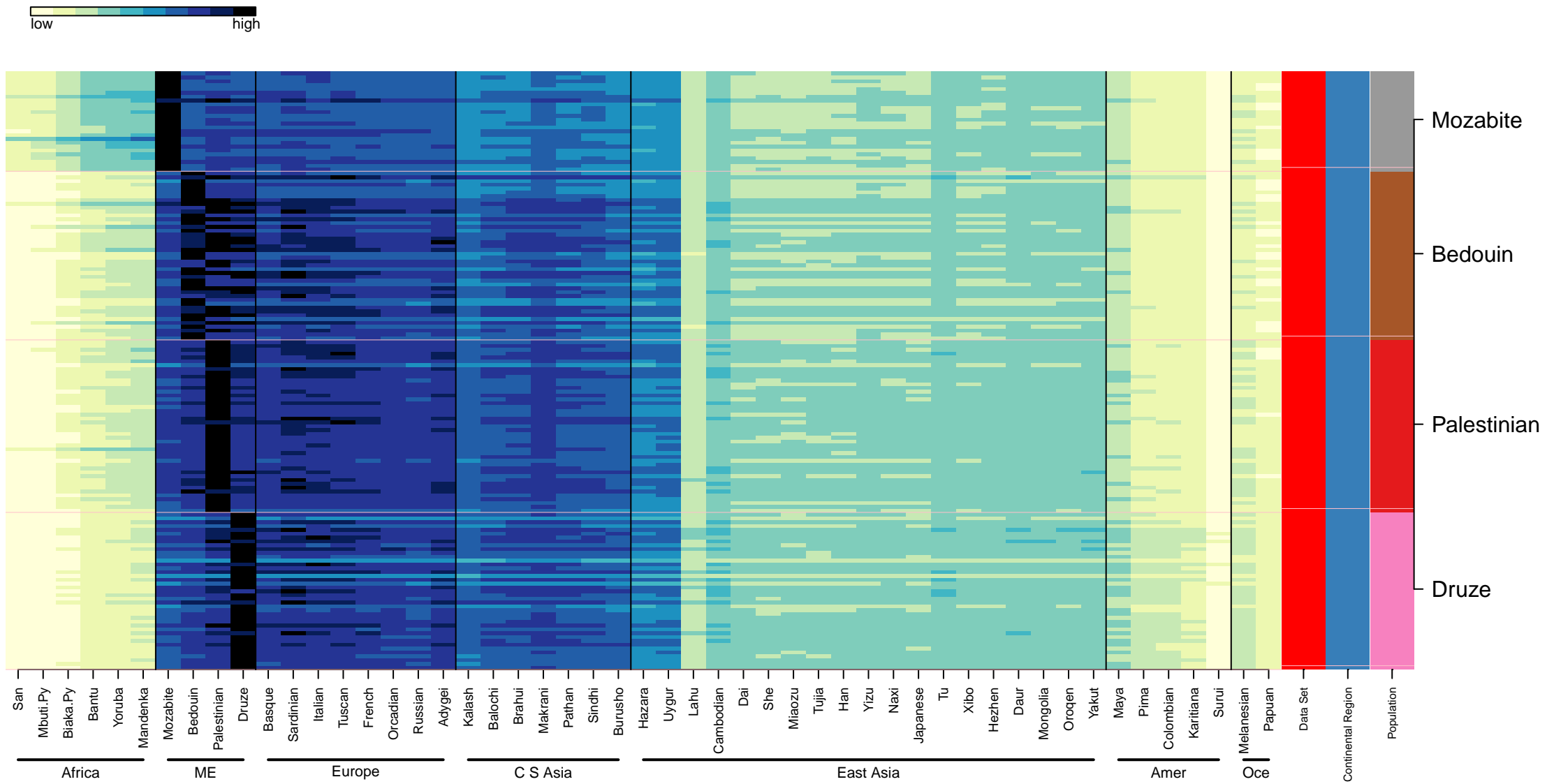

Supplement: Figure S4 — AMids of Middle East and North Africa. The Mozabites from Algeria, North Africa are the most similar to all sub-Saharan Africa, even if they have large differences to the San and the Mbuti Pygmies and higher European than African AMIds. Mozabite are isolated as indicated by the high values for their own Mozabite reference, which could also reflect a bias in HGDP, as Mozabite are the only representative of North Africa and this negatively impacts Ancestry Mapper results for this region. The Bedouins are much less similar to Africa than the Mozabite. They have few high AMids, show similarity to European references and heterogeneity. Druze and Palestinians show several high AMids and are closer to the European references than to the Mozabite. (PDF) [file pone.0049438.s004.pdf]

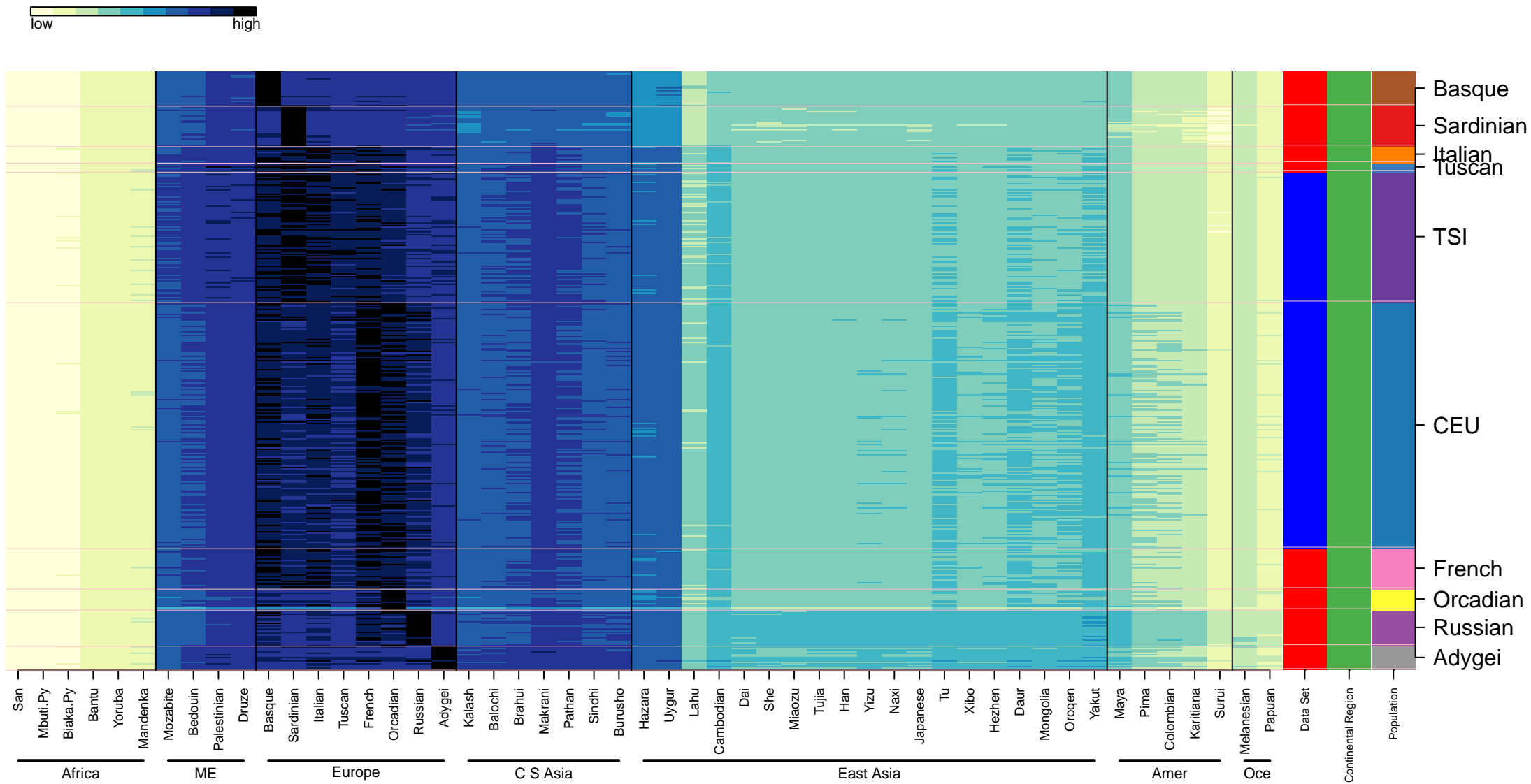

Supplement: Figure S5 — AMids of Europe. Many populations show several high European AMids, with Sardinians, Basques and Adygey the most isolated. The barcode makes each population unique. Russians and Adygey have strong East Asian AMids, which correlates with their geographical position between Europe and Asia. Italians are similar to Tuscans (both TSI/HapMap and Tuscans/HGDP) and it is difficult to distinguish between the two populations, with individuals showing high AMids for Italian, Sardinian and Tuscan. The third Italian population, from the isle of Sardinian, is clearly distinguishable and much more homogeneous, with high Sardinian and lower CSA/ME AMids. The HapMap CEU group shows heterogeneity, with Basque, French and Orcadian AMids producing distinctive barcodes that differentiates between individuals. (PDF) [file pone.0049438.s005.pdf]

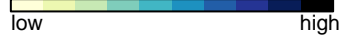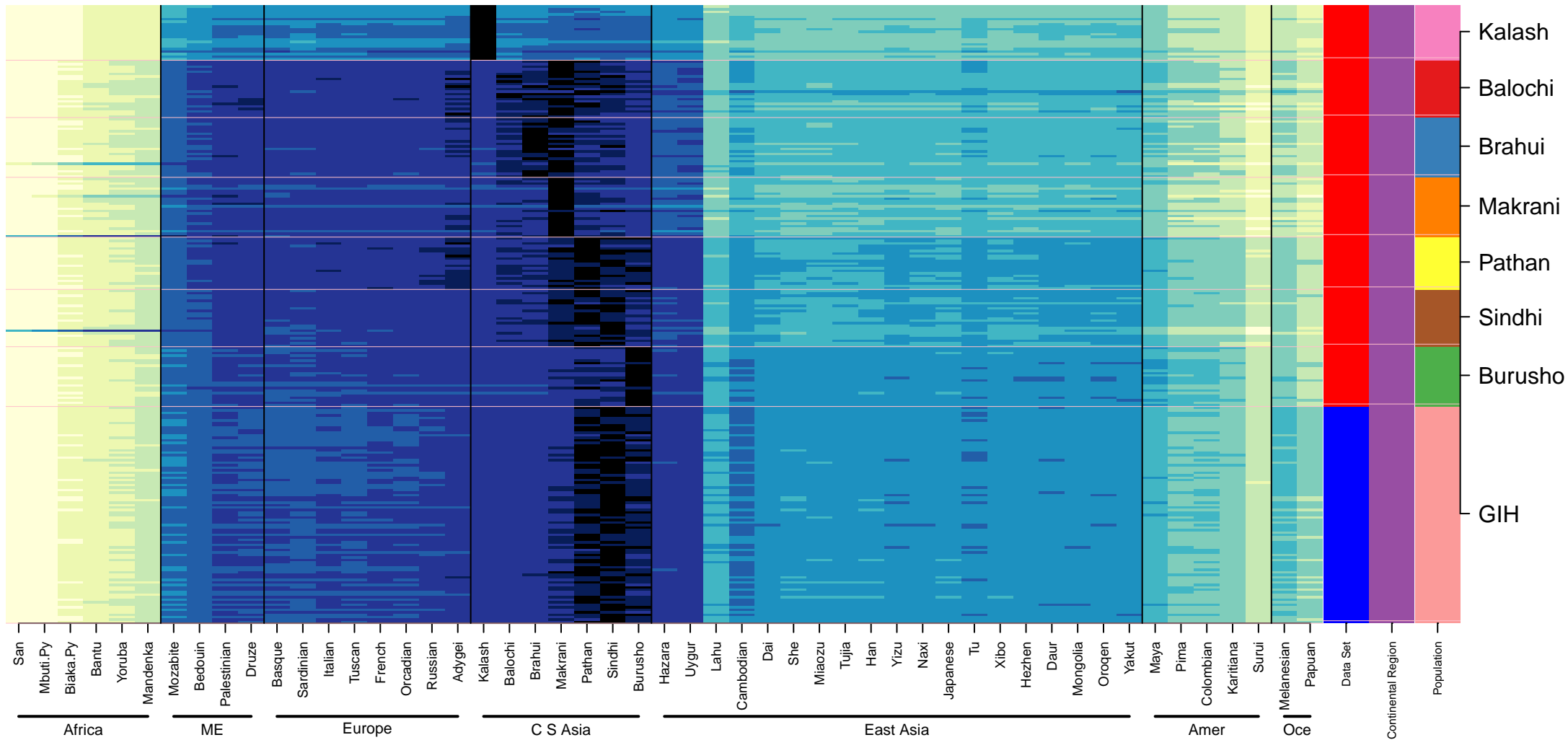

Supplement: Figure S6 — AMids of Central South Asia. Many populations show several high AMids, reflecting the many influences and migrations to and from CSA. The Kalash show isolation and are as similar to Europe as to CSA. The Makrani and the Balochi have a high level of intra-population heterogeneity. The EA references distinguish between the populations (e.g., Balochi/Brahui/Makrani have much lower EA AMids than Pathan/Sindhi/Burusho). Although Balochi, Makrani and Brahui live in the same region, Balochistan in Pakistan, differences can be identified. Brahui have stronger Brahui references. The Makrani have high levels of Brahui, Balochi and Makrani, and are heterogeneous with many individuals having different barcodes, but with the strongest influence being their own Makrani reference. The Balochi show even greater heterogeneity, with equally high coordinates for Balochi, Brahui and Makrani. Basque and Burusho languages have some similarity, but our data doesn’t show genetic similarity (supporting Ayub et all [48]). Two individuals (Makrani and Burushi) show high African AMids, possibly due to recent admixture, as previously reported [19]. (PDF) [file pone.0049438.s006.pdf]

low high

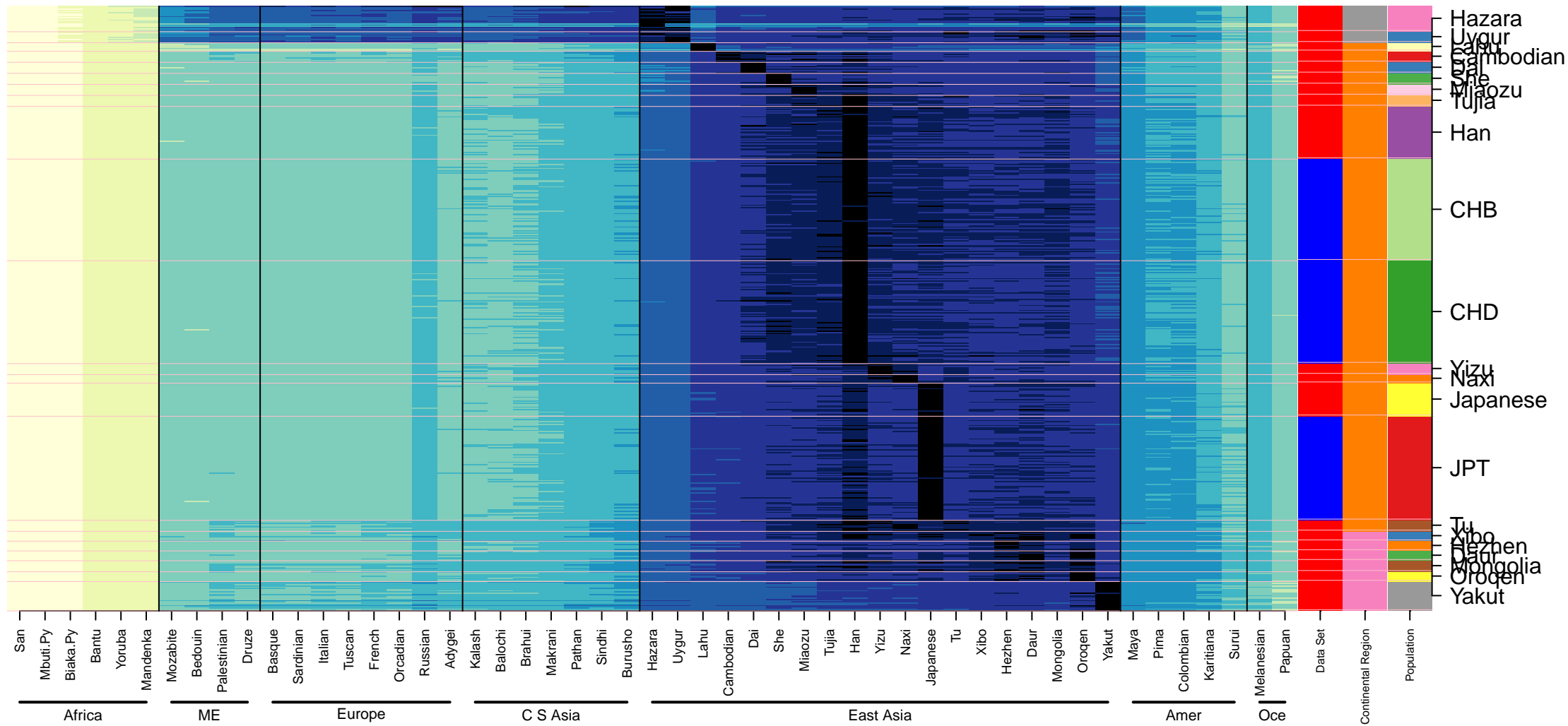

Supplement: Figure S7 — AMids of East Asia. We distinguish several blocks according to the population barcodes, which follow a South/North gradient: Hazara/Uygur, Lahu/Cambodian/Dai/She/Miaozu, Tujia/Han/Yizu/Naxia, Japanese/Tu, Xibo/Mongolia/Hezhen/Daur/Oroqen, and Yakut. The Hazaran and Uygur show similarities to both CSA and EA. Hazara and Uygur are close to Northern EA AMids; Hazarian history mentions a link to the Mongolians, which has been confirmed in two genetic studies [27] [49]. We don’t see similarities of the Hazara with other Pakistanis, confirming previous studies [26]. The ancestry of Uygur is uncertain: some studies show a stronger European influence [50] and others a stronger EA influence [51]. Our results support the EA influence, although Uygur are closer to Europe than the other Eastern Asians, which might explain the conflicting reports. Several groups have several high AMids, especially the Northern populations. The three Han datasets are similar, as are the two Japanese. The Yakut and the Lahu are the most isolated. The Oroquen, although geographically the closest to the Yakut, show weak genetic similarity with them. (PDF) [file pone.0049438.s007.pdf]

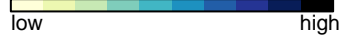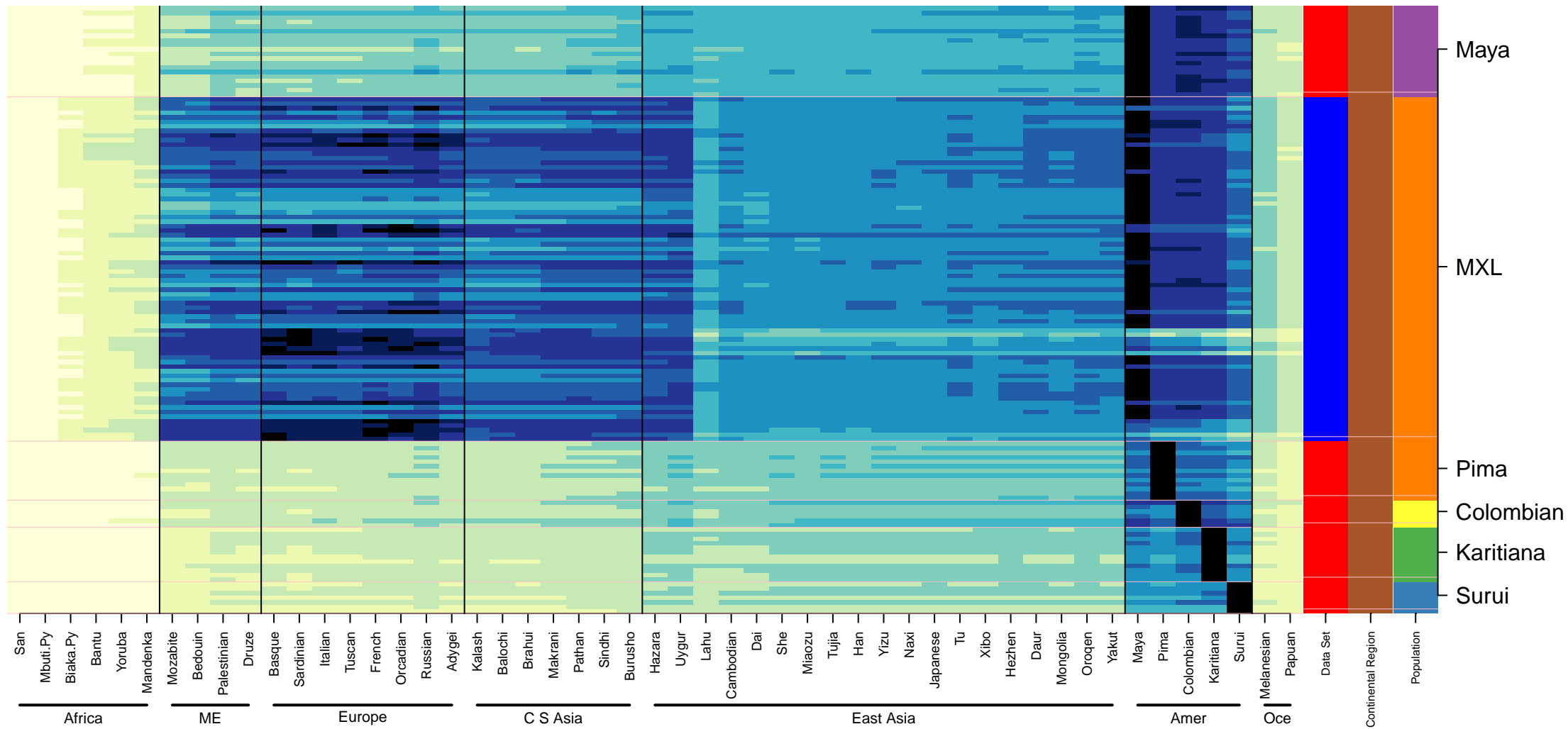

Supplement: Figure S8 — AMids of the Americas. The HapMap MXL population is more heteregeneous than the Native American populations. The Maya also has more high AMids than the other populations, indicating that they have the highest diversity and gene flow. The Surui population is quite isolated, being the most distant to many populations in the whole genomic map. Northern East Asia AMids are relatively high in Maya, probably reflecting the origin of the settlers of the American continent. (PDF) [file pone.0049438.s008.pdf]

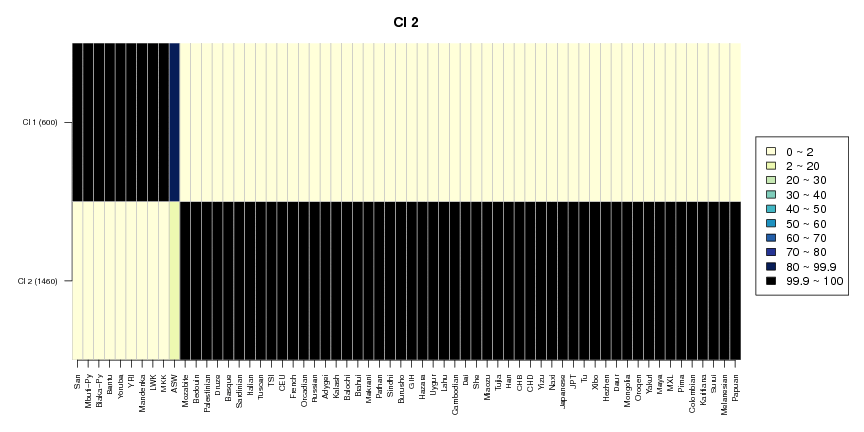

Supplement: Movie S1 — Animated GIF Figure For HapMap/HGDP clustering, fast. (GIF) [file pone.0049438.s013.gif]

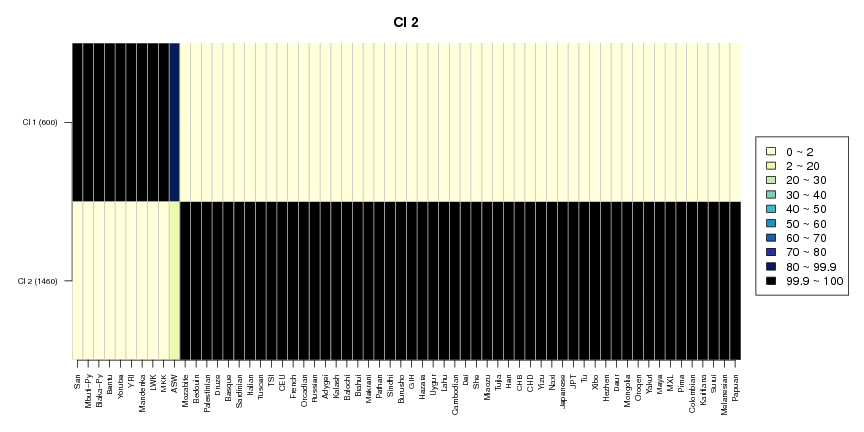

Supplement: Movie S2 — Animated GIF Figure For HapMap/HGDP clustering, slow. (GIF) [file pone.0049438.s014.gif]
