# Supplementary material for: HGDP and HapMap Analysis by Ancestry Mapper Reveals Local and Global Population Relationships
Source: PLoS One. 2012 Nov 26;7(11):e49438. doi: 10.1371/journal.pone.0049438 (PMC3506643; doi:10.1371/journal.pone.0049438)
Supplement: Figure S9 — AMids of Oceania. The Papuan and the Melanesians are quite isolated, as shown by high AMids for their own references and low AMids for all others. Papuan and Melanesian AMids are high for the other Oceanic reference, with the lowest AMids for Africa. Melanesians have relatively high AMids for the South East Asia references (Cambodian, Dai, Naxi, Yizu and Han), probably reflecting the settlement of Oceania from individuals coming from South East Asia. (PDF) [file pone.0049438.s009.pdf]

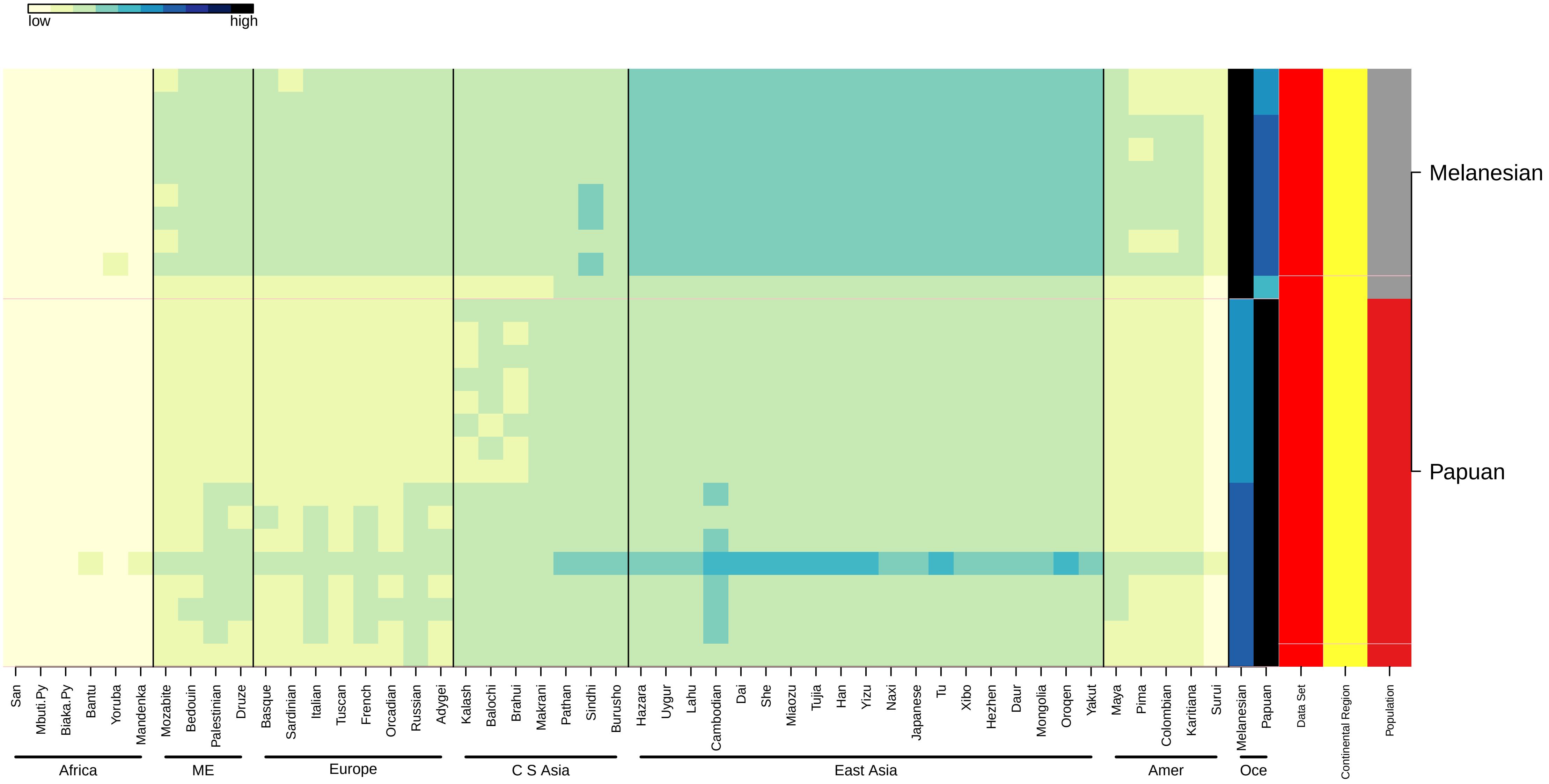
low
high
Melanesian
Papuan
San
Mbuti.Py
Biaka.Py
Bantu
Yoruba
Mandenka
Mozabite
Bedouin
Palestinian
Druze
Basque
Sardinian
Italian
Tuscan
French
Orcadian
Russian
Adygei
Kalash
Balochi
Brahui
Makrani
Pathan
Sindhi
Burusho
Hazara
Uygur
Lahu
Cambodian
Dai
She
Miaozu
Tujia
Han
Yizu
Naxi
Japanese
Tu
Xibo
Hezhen
Daur
Mongolia
Oroqen
Yakut
Maya
Pima
Colombian
Karitiana
Surui
Melanesian
Papuan
Data Set
Continental Region
Population
Africa
ME
Europe
C S Asia
East Asia
Amer
Oce
